# Development of an H_2_S-Associated Matrix Based on *Rhizostoma pulmo* Jellyfish Collagen: A Pilot Evaluation of Neuroprotective Effects and Cx43/p53 Regulation in Penetrating Traumatic Brain Injury

**DOI:** 10.3390/ijms27115134

**Published:** 2026-06-05

**Authors:** Stanislav Rodkin, Maria Kaplya, Sergey Golovin, Evgeniya Kirichenko, Chizaram Nwosu, Aleksandr Logvinov, Alina Sereda, Yulia Gordeeva, Aleksandr Romanov, Stanislav Bachurin

**Affiliations:** Research Laboratory “Medical Digital Images Based on the Basic Model”, Department of Bioengineering, Institute of Living Systems, Don State Technical University, Rostov-on-Don 344000, Russiabachurin.rostgmu@gmail.com (S.B.)

**Keywords:** traumatic brain injury (TBI), collagen scaffold, *Rhizostoma pulmo*, hydrogen sulfide (H_2_S), sodium thiosulfate, connexin 43 (Cx43), p53, neuroprotection, astroglial response, molecular dynamics simulation, biomaterials, central nervous system regeneration

## Abstract

Severe traumatic brain injury (TBI) is one of the leading causes of mortality and disability worldwide. To date, there are no clinically effective neuroprotective agents. Biomaterials that combine structural support for damaged tissue with a depot for therapeutic agents may represent a key solution to this problem. To evaluate the neuroprotective potential of a collagen matrix derived from the jellyfish *Rhizostoma pulmo* (*R. pulmo*) and modified with sodium thiosulfate (Na_2_S_2_O_3_) as an hydrogen sulfide (H_2_S) donor in a bioengineered platform for the treatment of severe TBI. Comprehensive characterization of the collagen matrix (electrophoresis, fluorescence microscopy), its implantation in a mouse model of severe TBI, and subsequent morphological, histological, ultrastructural, and immunohistochemical analyses of connexin 43 (Cx43) and p53 protein (p53) were performed. In addition, molecular dynamics simulations of the interactions between sulfur-containing compounds and target proteins were conducted. The effects were compared with inhibition of endogenous H_2_S synthesis using aminooxyacetic acid (AOAA). The collagen matrix retains the properties of type I collagen and forms a three-dimensional porous structure with high hydrophilicity and biocompatibility. Implantation ensures effective defect filling, reduces cystic degeneration, and preserves cortical structure. Modification with Na_2_S_2_O_3_ results in a significant reduction in both nuclear and cytoplasmic accumulation of p53, prevention of Cx43 dysregulation, a decrease in the proportion of damaged neurons and inflammatory infiltration, and preservation of tissue ultrastructure. In contrast, inhibition of CBS with AOAA exacerbates pathological changes. Molecular modeling demonstrated that S_2_O_3_^2−^ is capable of forming stable electrostatic interactions with domains of p53 and Cx43 under conditions of acidosis and elevated Ca^2+^. A collagen matrix derived from *R. pulmo* and modified with Na_2_S_2_O_3_ represents a promising biodegradable platform that combines structural support with local H_2_S-dependent regulation of key mechanisms of secondary brain injury. This approach provides a multilevel neuroprotective effect and opens new opportunities for the development of therapeutic implants for severe TBI.

## 1. Introduction

Traumatic brain injury (TBI) is the most common traumatic damage to the central nervous system (CNS) and is a frequent cause of death and disability worldwide. Severe TBI, particularly of a penetrating type, often results in immediate tissue damage, hemorrhage, and a rapidly propagating wave of secondary injury processes, including inflammation, oxidative stress, edema, and apoptosis, ultimately leading to severe cognitive and motor impairments [1]. The problem is compounded by the lack of clinically effective neuroprotective agents capable of protecting neurons and glial cells within the lesion area. Current therapeutic strategies are primarily focused on stabilization and subsequent rehabilitation but do not provide approaches to stimulate regeneration of damaged neurons or reduce secondary injury [2]. Therefore, innovative approaches to TBI treatment may be key to the development of next-generation neuroprotective therapies.

Biomaterials, such as collagen scaffolds, have emerged as promising tools for CNS tissue engineering, providing structural support, biocompatibility, and a favorable environment for enhanced cell migration and regeneration [3]. Marine-derived collagen, particularly from jellyfish such as *Rhizostoma pulmo* (*R. pulmo*), offers sustainable alternatives to mammalian sources, demonstrating high yield, antioxidant properties, and excellent cytocompatibility [4]. Such collagen has shown potential in biomedical applications, including acceleration of wound healing [5], as well as serving as scaffolds for cell adhesion [6], making it suitable for implantation into brain injury sites. A promising direction is the development of hybrid collagen-based matrices incorporating molecular neuroprotective agents [7], among which hydrogen sulfide (H_2_S) donors are of particular interest.

H_2_S, an endogenous gasotransmitter, plays a key neuroprotective role in TBI by reducing brain edema, oxidative stress, apoptosis, and autophagy, as well as improving motor and cognitive outcomes. The key enzyme responsible for H_2_S biosynthesis in nervous tissue is cystathionine-β-synthase (CBS). H_2_S biosynthesis in the brain represents a dynamic system that rapidly responds to stress stimuli, including traumatic injury such as TBI. Maintenance of physiological H_2_S levels is essential for normal molecular and cellular processes [8]. At present, a wide range of H_2_S donors exists, ranging from simple inorganic salts to complex hybrid systems [9]. However, none of these donors has yet received full approval for routine clinical use in the treatment of nervous system-related pathologies, including TBI. Sodium thiosulfate (STS), a clinically approved agent with detoxifying and antihistamine properties, is considered a potential source of H_2_S and may exert H_2_S-associated biological effects [10]. In contrast, inhibitors of H_2_S biosynthesis, such as aminooxyacetic acid (AOAA), target CBS and allow investigation of the possible role of H_2_S-dependent signaling mechanisms in response to various damaging stimuli [11]. Disruption of intracellular H_2_S levels is a key factor that exacerbates neurodegenerative processes [8].

TBI is associated with impaired intercellular communication, enhanced inflammation, and the development of reactive gliosis, making the study of gap junction proteins, particularly connexin 43 (Cx43), especially relevant. This protein is widely expressed in astrocytes and plays a key role in regulating intercellular exchange of ions and metabolites, as well as in the propagation of injury signals within brain tissue. Alterations in the expression and spatial localization of Cx43 are associated with neuroinflammation and secondary neural tissue damage [12]. An important molecular marker of the cellular response to injury is the transcription factor p53, known as the “guardian of the genome”. Under conditions of neurotrauma, activation of p53 is closely associated with the initiation of programmed cell death, as this protein serves as a key regulator of apoptotic signaling pathways in response to DNA damage, oxidative stress, and mitochondrial dysfunction. Increased p53 expression following TBI is associated with apoptosis activation, disruption of mitochondrial permeability, and exacerbation of secondary neuronal injury. Moreover, the subcellular localization of p53 plays a critical role in its functional activity: nuclear accumulation is associated with transcriptional activation of pro-apoptotic genes, whereas the cytoplasmic fraction of p53 may participate in transcription-independent regulation of mitochondrial pathways of cell death.

In our recent study, collagen from *R. pulmo* jellyfish was isolated and characterized, and three-dimensional matrices were developed using lyophilization, exhibiting a pronounced porous structure and favorable properties for tissue engineering applications [13]. Previously, we also investigated in detail the dynamics of expression of key proteins involved in the cellular response to injury, including Cx43 [14,15] and p53, in an experimental TBI model. In addition, a series of studies demonstrated the important role of H_2_S in regulating the expression of proteins involved in apoptosis control, including p53, under conditions of traumatic injury to nervous tissue [16,17,18].

The present study represents a logical continuation of these fundamental investigations and is aimed at exploring the possibility of integrating previously obtained data into an applied approach involving the development of an H_2_S-associated collagen matrix based on *R. pulmo*, capable of combining structural support for damaged tissue with potential modulation of molecular mechanisms underlying secondary brain injury.

In this study, we investigated the feasibility of using collagen matrices derived from *R. pulmo* jellyfish as carriers for sulfur-containing compounds with potential H_2_S-associated effects in a mouse model of severe penetrating TBI. The collagen matrices were used in their native form, as well as in combination with STS as a potential H_2_S donor or with the inhibitor of its synthesis, AOAA. A comprehensive evaluation of the biocompatibility and neuroprotective properties of the developed matrix was performed, including analysis of morphological changes in brain tissue, assessment of the expression and subcellular localization of Cx43 and p53, and molecular dynamics modeling of the interactions between these proteins and sulfur-containing ligands. The obtained results may contribute to the development of novel biomaterials for TBI treatment that combine structural support of damaged tissue with potential modulation of molecular mechanisms of secondary brain injury.

## 2. Results

### 2.1. Analysis of Collagen Composition from Rhizostoma pulmo Jellyfish by SDS-PAGE

**Figure 1:**
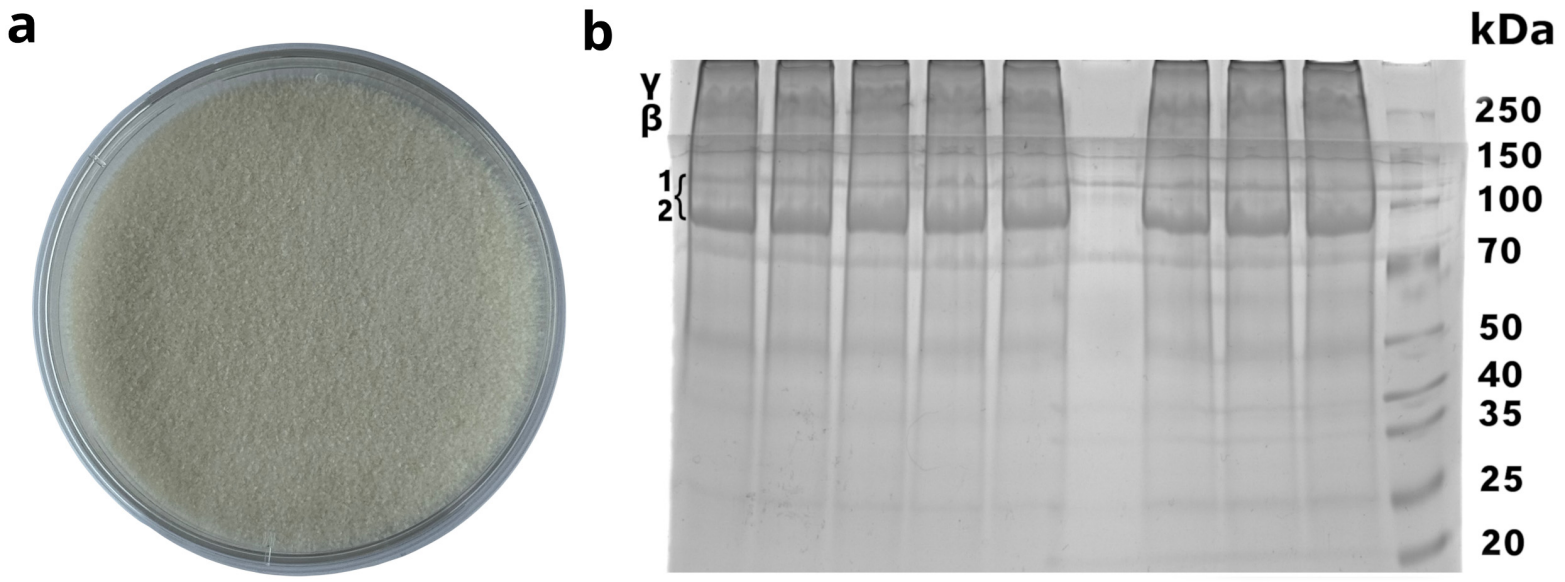
Characterization of lyophilized collagen from *R. pulmo*. (**a**) Lyophilized collagen powder from *R. pulmo*. (**b**) SDS-PAGE electrophoretic profile of lyophilized *R. pulmo* collagen samples: the molecular weight marker is shown on the left; characteristic bands corresponding to the γ-component (trimer of α-chains), β-component (dimer of α-chains), as well as individual α1- and α2-chains of collagen were identified.

Lyophilized collagen obtained from the jellyfish *R. pulmo* appeared as a homogeneous, finely dispersed white powder without visible mechanical impurities (Figure 1a), indicating preservation of protein structure and suitability of the material for further experimental use.

According to SDS-PAGE analysis, the submolecular fraction of jellyfish collagen exhibited a pattern typical of fibrillar collagen, with the presence of two α-chains and a dimeric fraction. The most intense band, with a molecular weight of approximately 95–100 kDa, was clearly visualized and interpreted as the α1 chain, whereas a less pronounced band in the region of approximately 105 kDa corresponded to the α2 chain. In the high molecular weight region, a dimeric fraction corresponding to the β-chain was detected. The diffuse appearance of the major bands is characteristic of jellyfish collagen and is due to the fibrillar nature of the protein, as well as the specific interaction of the triple helix with sodium dodecyl sulfate, resulting in electrophoretic mobility that does not fully correspond to the molecular weight of marker proteins (Figure 1b).

### 2.2. Fluorescence Analysis of Collagen Sponge Morphology

The morphology of collagen sponges derived from *R. pulmo* jellyfish was analyzed to assess their suitability as three-dimensional matrices for biomedical applications. The collagen sponges were stained with the fluorescent dyes Hoechst 33342, propidium iodide, and SYTOX Green.

**Figure 2:**
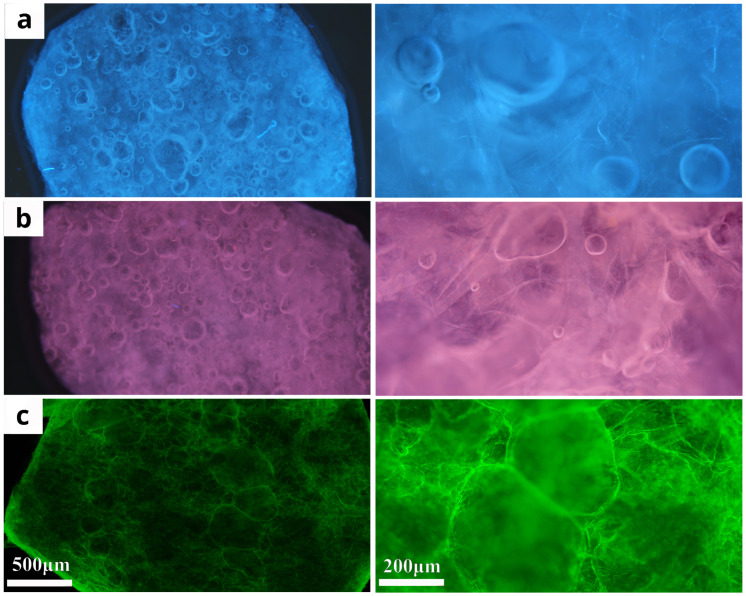
Fluorescence microscopy of collagen matrices. The image shows matrices derived from *R. pulmo* collagen stained with: (**a**) Hoechst 33342, (**b**) Propidium iodide, and (**c**) SYTOX Green. Scale bars: 500 and 200 μm.

The obtained fluorescence images demonstrated a well-defined three-dimensional porous architecture of the collagen sponges, with a system of interconnected pores uniformly distributed throughout the matrix volume, which determines the high absorption capacity of the material and its large internal surface area (Figure 2). It should be noted that under transmitted light, the surface and structure of the collagen sponge are barely distinguishable, whereas the use of hydrophilic fluorescent dyes, which distribute within the pore space, allows clear visualization of the pore network and septa. The applied fluorescence staining method provides rapid and informative visualization of the porous structure of the collagen scaffold without the need for scanning electron microscopy, making it a convenient approach for morphological assessment and quality control of the formed matrices.

In our previous studies on the swelling behavior of collagen sponges, it was demonstrated that the samples exhibit a high degree of water absorption at pH 5.0 and pH 8.0. Analysis of swelling kinetics revealed pronounced hydrophilic properties of the collagen matrix [13], which is consistent with the observed three-dimensional porous organization and high water-retention capacity of the material.

### 2.3. Modeling of Severe TBI and Evaluation of Tolerance to Collagen Matrix Implantation

**Figure 3:**
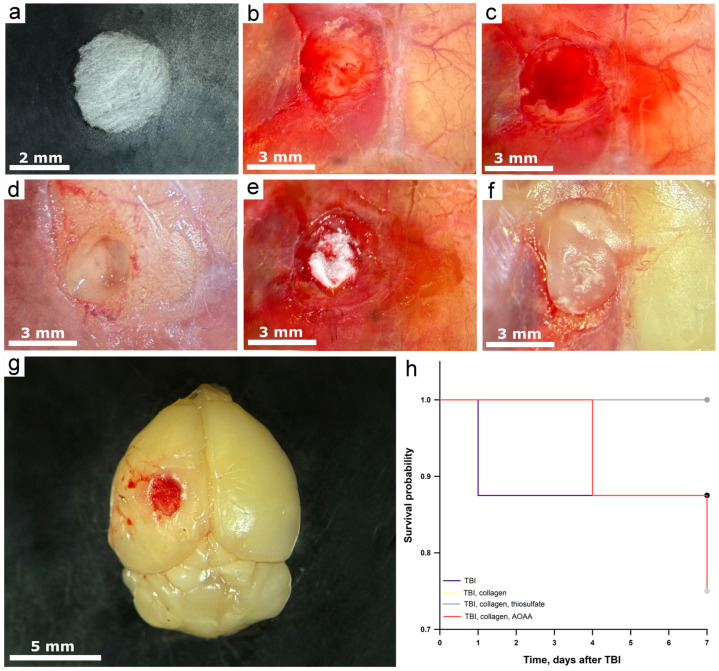
Modeling of traumatic brain injury with formation of an extensive defect and implantation of a collagen matrix. (**a**) Round collagen matrix with a diameter of 3 mm. (**b**) Formation of a trephination opening using a dental drill. (**c**) Application of a controlled cortical impact resulting in tissue defect formation. (**d**) Irrigation of the wound surface with sterile saline. (**e**) Implantation of the collagen matrix into the injury site. (**f**) Sealing of the bone defect using bone wax. (**g**) Gross brain specimen with reproduced TBI at day 1. Scale bars: (a) 2 mm; (b–f) 3 mm; (g) 5 mm. (**h**) Kaplan–Meier survival curves over 7 days after TBI modeling. Comparison of groups: TBI, TBI + collagen matrix, TBI + collagen matrix loaded with Na_2_S_2_O_3_, and TBI + collagen matrix loaded with AOAA. The survival curves of the TBI + collagen matrix and TBI + collagen matrix loaded with Na_2_S_2_O_3_ groups were superimposed throughout the 7-day observation period. Statistical analysis was performed using the log-rank test; differences between groups were not statistically significant (*p* = 0.283). Each experimental group included *n* = 8 animals.

In all animals, induction of a severe TBI model in mice with the formation of a trephination opening followed by impact using a freely falling weight consistently resulted in the formation of a focal defect in the cerebral cortex. To close the formed defect, a round collagen matrix with a diameter of 3 mm was used (Figure 3a), characterized by a pronounced porous structure with multidirectional fibrillar strands. The trephination opening was created using a dental drill (Figure 3b), after which a controlled impact was applied to the cortex using a freely falling weight, leading to the formation of a tissue defect (Figure 3c). Prior to implantation, the wound surface was irrigated with sterile saline until removal of blood clots and tissue debris. The image shows an intensively irrigated wound obtained from a mouse head specimen after decapitation to improve visualization of the injury site (Figure 3d). The collagen matrix was then placed into the formed cavity, conforming to the geometry of the wound channel and tightly filling the lesion area (Figure 3e). The final stage of the procedure involved sealing the bone defect using bone wax (Figure 3f).

In the early post-traumatic period, a well-defined cavity was observed at the site of impact, with focal hemorrhages at the periphery and moderate hyperemia of the surrounding soft tissues, consistent with previously described experimental models of severe TBI in mice based on the freely falling weight method. Macroscopic examination of the brain at the analyzed time points revealed a localized area of damage corresponding to the impact site (Figure 3g). At the periphery of the defect, areas of pronounced glial response and developing scar tissue were observed, representing a typical morphological pattern for experimental TBI models in rodents. In animals without matrix implantation, the post-traumatic lesion showed a tendency toward the formation of a more pronounced cystic cavity with thinning of the adjacent cortex, whereas in the group with the collagen scaffold, the cavity was largely filled with the implant and granulation tissue. A more detailed characterization of cellular changes, the extent of glial response, and inflammatory infiltration is presented in the section dedicated to histological analysis of brain tissue.

Following implantation of collagen matrices into the injury site, most animals tolerated the procedure well. The general condition of the mice remained stable, and no signs of severe clinical deterioration were observed. During the 7-day observation period, three deaths were recorded: one in the TBI group on day 1 and two in the TBI group with collagen matrix loaded with AOAA on days 4 and 7. No deaths were observed in the groups with implantation of native collagen matrix or collagen matrix loaded with sodium thiosulfate (Na_2_S_2_O_3_).

Kaplan–Meier survival analysis showed no statistically significant differences between the experimental groups (log-rank test, χ^2^ = 3.81; *p* = 0.283); however, a trend toward reduced survival was observed in the group treated with AOAA-loaded collagen matrix (75% compared to 100% in the collagen and collagen + Na_2_S_2_O_3_ groups, and 87.5% in the TBI group) (Figure 3h). The lack of statistical significance is likely due to the small sample size, which does not allow definitive conclusions at this stage regarding the effect of the studied compounds on procedural tolerance and post-injury outcomes.

These findings demonstrate the reproducibility of the severe TBI model and confirm the feasibility of using a collagen matrix to fill post-traumatic defects. However, further studies with larger sample sizes are required to clarify the impact of pharmacological modification of the matrix on survival rates and the course of post-traumatic recovery.

### 2.4. Morphological Changes in Brain Tissue Following TBI

**Figure 4:**
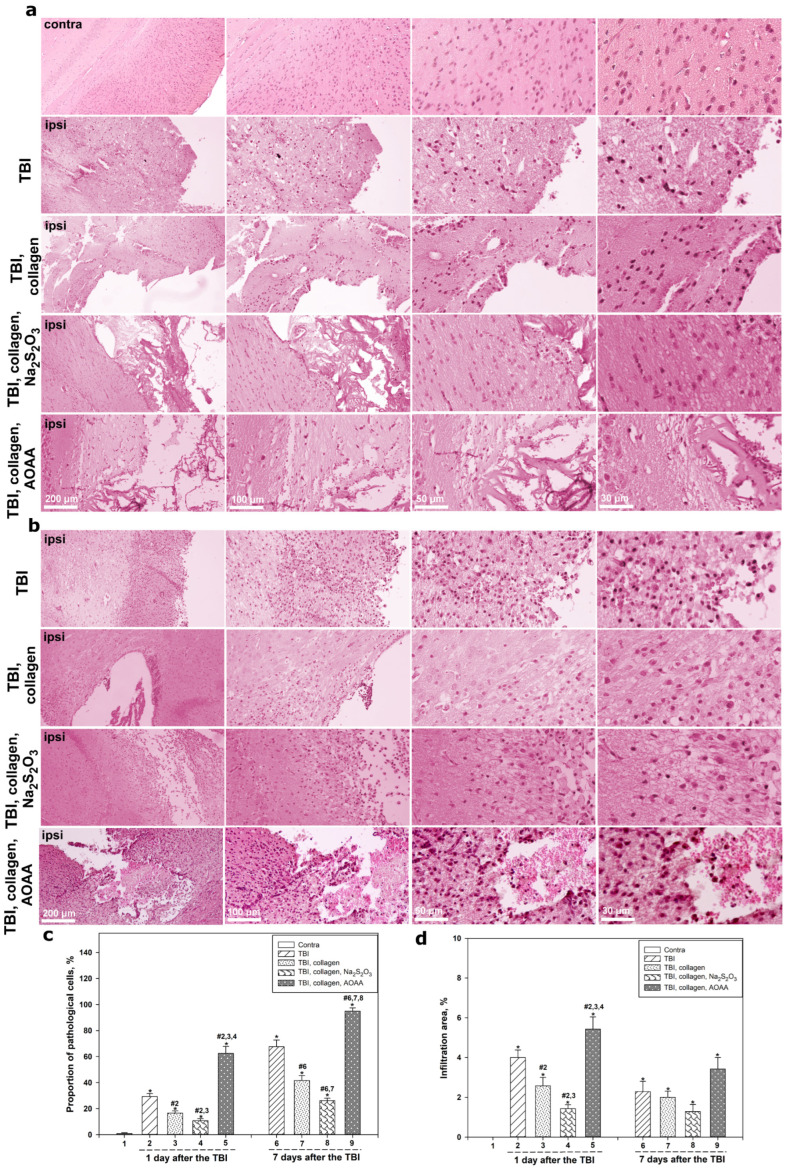
Morphological assessment of brain tissue damage and inflammatory infiltration following TBI (hematoxylin and eosin staining). (**a**) Micrographs of cerebral cortex sections 1 day after TBI. (**b**) Micrographs of cerebral cortex sections 7 days after TBI. (**c**) Proportion of pathologically altered cells in the lesion area, expressed as a percentage of the total number of cells. (**d**) Area of infiltration, expressed as a percentage of the total analyzed tissue area. Groups: Contra—contralateral cortex; TBI—ipsilateral cortex 1 day after TBI; TBI + collagen—TBI with collagen matrix implantation; TBI + collagen + Na_2_S_2_O_3_—TBI with collagen matrix loaded with sodium thiosulfate; TBI + collagen + AOAA—TBI with collagen matrix under CBS inhibition (AOAA). In panel (d), the Contra bar overlaps with the baseline due to the near-zero infiltration area in the contralateral hemisphere. For panels (**c**–**d**), the first five bars correspond to day 1 after TBI, and the subsequent four bars correspond to day 7 after TBI. * *p* < 0.05—statistically significant differences relative to the contralateral hemisphere; # *p* < 0.05—statistically significant differences relative to ipsilateral hemispheres of experimental groups. Significance markers #2, #3, #4, #6, #7, and #8 above bars indicate statistically significant differences between corresponding groups. Data are presented as mean ± SEM (*n* = 6 per group). Statistical analysis was performed using one-way ANOVA followed by the Newman–Keuls post hoc test.

TBI induced pronounced morphological changes in the cerebral cortex, characterized by the development of degenerative processes in neurons, disruption of neuropil structure, and an increase in the number of glial cells within the lesion area. As early as 1 day after TBI, signs of acute tissue damage were observed, including the appearance of cells with pathological morphology, rarefaction of the neuropil, and foci of inflammatory infiltration (Figure 4a).

In the groups treated with the collagen matrix, fragments of collagen fibrils were visualized in the lesion area 1 day after injury, most clearly detected in samples with Na_2_S_2_O_3_ and AOAA. In some cases, partial displacement or loss of individual collagen matrix fragments from the section plane was observed, which is likely associated with sample preparation procedures and the structural density of the material. The most pronounced structural changes were recorded 7 days after injury (Figure 4b), indicating progression of post-traumatic degenerative processes and development of secondary tissue damage. At 7 days after TBI, a decrease in the number of visualized collagen fragments was observed, which may indicate partial degradation or remodeling of the collagen matrix within brain tissue.

Quantitative analysis of the proportion of pathologically altered cells in the cerebral cortex after TBI revealed a pronounced phase-dependent dynamic of tissue damage. In the contralateral hemisphere, the proportion of pathological cells remained minimal. At 1 day after TBI, this parameter significantly increased in the ipsilateral cortex compared to the contralateral hemisphere (*p* < 0.05). Application of the collagen matrix resulted in a 43% reduction in the proportion of pathological cells relative to the TBI group (*p* < 0.05), whereas the use of collagen loaded with Na_2_S_2_O_3_ led to a 63% decrease compared to the TBI group (*p* < 0.05). Importantly, animals with implantation of the collagen matrix loaded with Na_2_S_2_O_3_ demonstrated a significantly lower proportion of pathologically altered cells compared to animals implanted with the native collagen matrix alone (*p* < 0.05), indicating an additional neuroprotective effect associated with incorporation of Na_2_S_2_O_3_ into the collagen scaffold. In contrast, inhibition of CBS using AOAA caused more than a twofold increase in the proportion of pathologically altered cells (*p* < 0.05) compared to the TBI group. Moreover, animals implanted with the AOAA-loaded collagen matrix demonstrated significantly higher levels of pathological cell alterations compared both to animals treated with the native collagen matrix and to animals implanted with the collagen matrix loaded with Na_2_S_2_O_3_ (*p* < 0.05), indicating aggravation of post-traumatic tissue damage under conditions of endogenous H_2_S synthesis inhibition (Figure 4c).

At 7 days after TBI, the proportion of pathological cells remained elevated in all experimental groups relative to the contralateral hemisphere (*p* < 0.05). In the TBI group, this parameter remained significantly increased compared to the contralateral cortex (*p* < 0.05). Application of the collagen matrix resulted in a 38% reduction in the proportion of pathological cells relative to the TBI group (*p* < 0.05), whereas Na_2_S_2_O_3_ reduced this parameter by 61% compared to the TBI group (*p* < 0.05). At this time point, animals implanted with the collagen matrix loaded with Na_2_S_2_O_3_ also exhibited a significantly lower proportion of pathological cells compared to animals treated with the native collagen matrix (*p* < 0.05), suggesting that pharmacological modification of the scaffold with Na_2_S_2_O_3_ further enhanced its protective effect during progression of secondary post-traumatic injury. The most pronounced morphological changes were observed in the TBI + AOAA group, where the proportion of pathologically altered cells increased more than 1.4-fold (*p* < 0.05) compared to the TBI group (Figure 4c).

Analysis of the infiltration area in the lesion zone also revealed marked changes in the early post-traumatic period. In the contralateral hemisphere, no signs of infiltration were observed. At 1 day after TBI, the infiltration area significantly increased relative to the contralateral hemisphere (*p* < 0.05), indicating a pronounced inflammatory response and vascular damage. Application of the collagen matrix resulted in a 33% reduction in infiltration area relative to the TBI group (*p* < 0.05), whereas collagen loaded with Na_2_S_2_O_3_ reduced this parameter by 63% (*p* < 0.05). In addition, implantation of the collagen matrix loaded with Na_2_S_2_O_3_ resulted in a significantly smaller infiltration area compared to implantation of the native collagen matrix alone (*p* < 0.05), indicating a more pronounced attenuation of inflammatory changes following pharmacological modification of the scaffold. In contrast, inhibition of CBS using AOAA led to a 42% increase in infiltration area (*p* < 0.05) compared to the TBI group (Figure 4d), reflecting more severe tissue damage. In addition, animals implanted with the AOAA-loaded collagen matrix demonstrated significantly greater infiltration areas compared both to animals treated with the native collagen matrix and to animals implanted with the collagen matrix loaded with Na_2_S_2_O_3_ (*p* < 0.05) (Figure 4d).

At 7 days after TBI, the infiltration area remained elevated in all experimental groups compared to the contralateral hemisphere (*p* < 0.05); however, no statistically significant differences between groups were observed (Figure 4d).

### 2.5. Effect of Collagen Matrices Modified with Na_2_S_2_O_3_ and AOAA on Cx43 Expression After TBI

**Figure 5:**
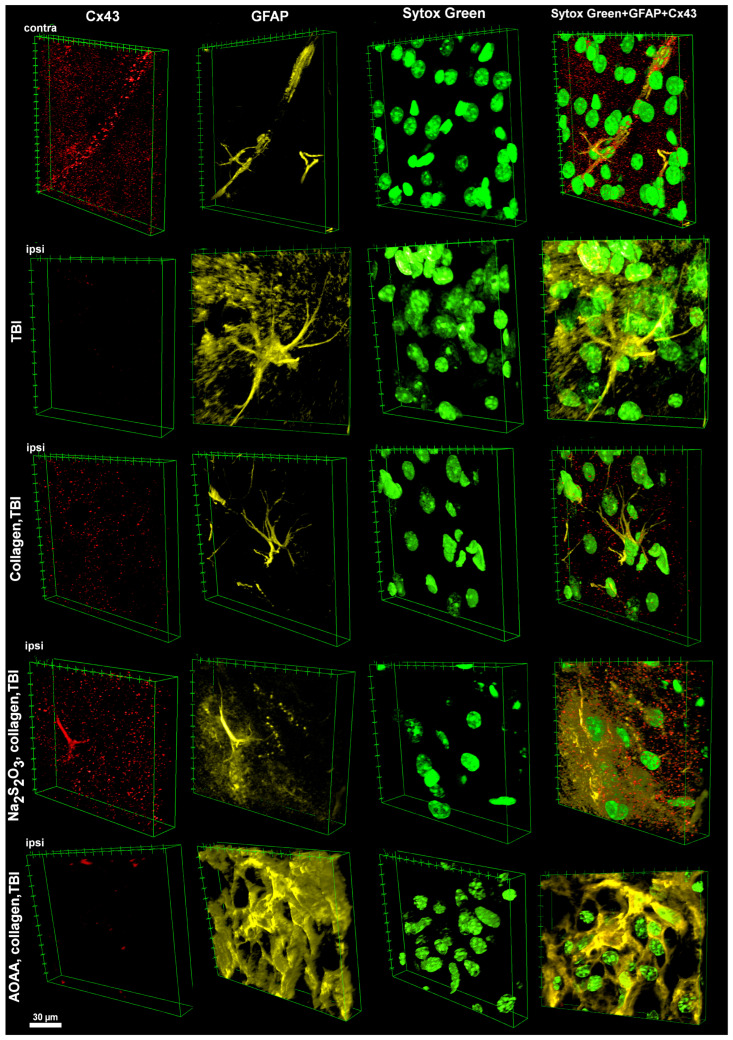
Confocal laser scanning microscopy characterizing Cx43 expression 1 day after TBI. Images of the contralateral and ipsilateral hemispheres of the brain from animals in the experimental groups are shown: TBI, TBI + collagen matrix, TBI + collagen matrix loaded with Na_2_S_2_O_3_, and TBI + collagen matrix loaded with AOAA. The red fluorescent signal corresponds to Cx43 immunolabeling, dark yellow to the astrocyte marker GFAP, and green to nuclear staining with SYTOX Green. Colocalization of markers is presented as merged signals with three-dimensional reconstruction of the final images. Scale bar: 30 μm.

In the contralateral hemisphere, a normal pattern of distribution of the studied markers was preserved at all observation time points. Cx43 expression was uniform, without the formation of aggregates or clusters. Astrocytes exhibited typical morphology with clearly distinguishable cell bodies and processes upon GFAP immunostaining; cell nuclei retained an intact structure without signs of fragmentation or chromatin condensation when stained with SYTOX Green (Figure 5).

At 1 day after TBI, a sharp decrease in Cx43 expression was observed in the ipsilateral hemisphere, up to an almost complete loss of immunoreactivity. At the same time, signs of reactive astrogliosis were detected, including increased GFAP signal intensity and altered astrocyte morphology. Astrocytic processes became thicker, locally shortened, and partially lost their clear orientation. SYTOX Green staining revealed nuclear damage, including deformation, fragmentation, and chromatin condensation (Figure 5).

Implantation of the collagen matrix exerted a moderate neuroprotective effect, manifested as a less pronounced decrease in Cx43 expression compared to the untreated TBI group. GFAP signal intensity remained elevated but was lower than in the TBI group. Morphological nuclear alterations persisted, although signs of fragmentation were less frequent. Loading the matrix with Na_2_S_2_O_3_ enhanced this effect, most notably reflected in the relative preservation of Cx43 levels and a less pronounced glial response (Figure 5).

In the group treated with the AOAA-loaded matrix, the opposite effect was observed. Cx43 levels remained critically low, comparable to the TBI group. GFAP expression was the highest among all groups and was accompanied by marked disorganization of astrocytic processes. In some regions, an intense diffuse GFAP signal not associated with clearly distinguishable processes was observed, likely reflecting disruption of astrocytic cytoskeletal integrity, fragmentation of processes, and possible release of Cx43 into the extracellular space due to cellular damage. More pronounced nuclear alterations were also observed (Figure 5).

**Figure 6:**
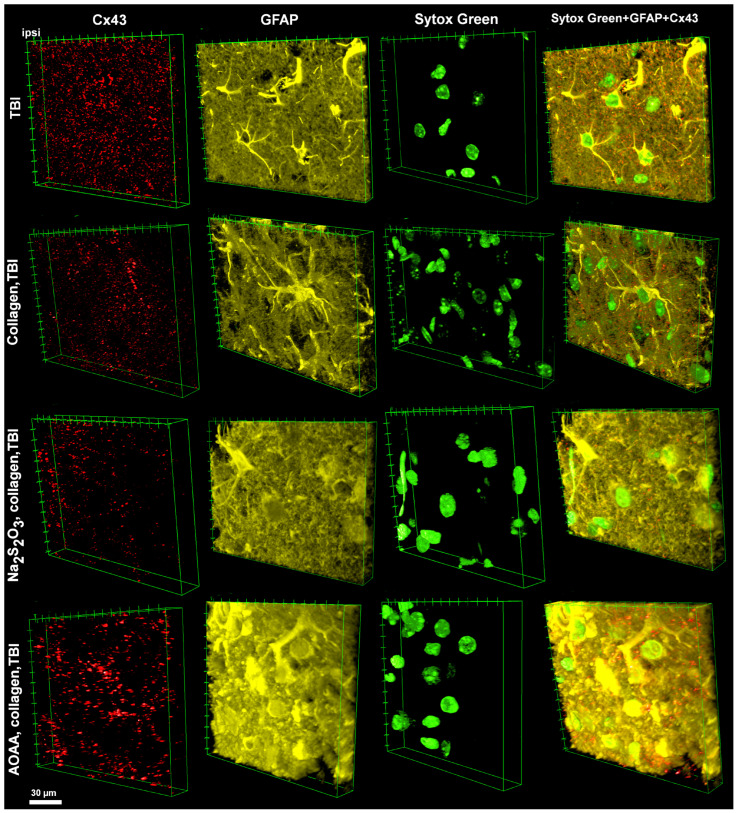
Confocal laser scanning microscopy characterizing Cx43 expression 7 days after TBI. Images of the contralateral and ipsilateral hemispheres of the brain from animals in the experimental groups are shown: TBI, TBI + collagen matrix, TBI + collagen matrix loaded with Na_2_S_2_O_3_, and TBI + collagen matrix loaded with AOAA. The red fluorescent signal corresponds to Cx43 immunolabeling, dark yellow to the astrocyte marker GFAP, and green to nuclear staining with SYTOX Green. Colocalization of markers is presented as merged signals with three-dimensional reconstruction of the final images. Scale bar: 30 μm.

At 7 days after TBI, a significant increase in Cx43 expression was observed in the ipsilateral hemisphere across all experimental groups; however, the extent and pattern of changes differed markedly. In the TBI group, a high level of Cx43 immunoreactivity was observed, with uneven signal distribution and the formation of aggregates of varying size. GFAP expression further increased compared to day 1, reflecting progression of reactive astrogliosis. Astrocytes exhibited pronounced hypertrophy, disruption of the spatial organization of their processes, and signs of partial fragmentation. Features of cell death, including nuclear deformation and fragmentation, persisted (Figure 6).

Implantation of the collagen matrix reduced the degree of Cx43 upregulation and decreased the extent of its aggregation compared to the TBI group. GFAP expression remained elevated; however, astrocyte morphology was less altered, and signs of process disorganization were less frequent. Morphological signs of nuclear damage were present but less pronounced. The combination of the matrix with Na_2_S_2_O_3_ further limited the severity of reactive changes (Figure 6).

In the AOAA group, these positive effects were abolished. The highest level of Cx43 expression was observed, accompanied by the formation of large aggregates and pronounced heterogeneity of signal distribution. GFAP expression was maximal among all groups and was associated with the most severe disorganization of astrocytic structure. In many regions, an intense diffuse GFAP signal lacking clear association with cellular processes was detected, which may indicate severe disruption of astrocyte integrity, fragmentation of processes, and release of Cx43 into the intercellular space as a result of cellular damage. Astrocytes frequently exhibited atypical morphology with shortened or poorly visualized processes. Pronounced nuclear fragmentation persisted, indicating ongoing cellular damage (Figure 6).

### 2.6. Quantitative Assessment of Cx43 Fluorescence Intensity and Aggregation After TBI

**Figure 7:**
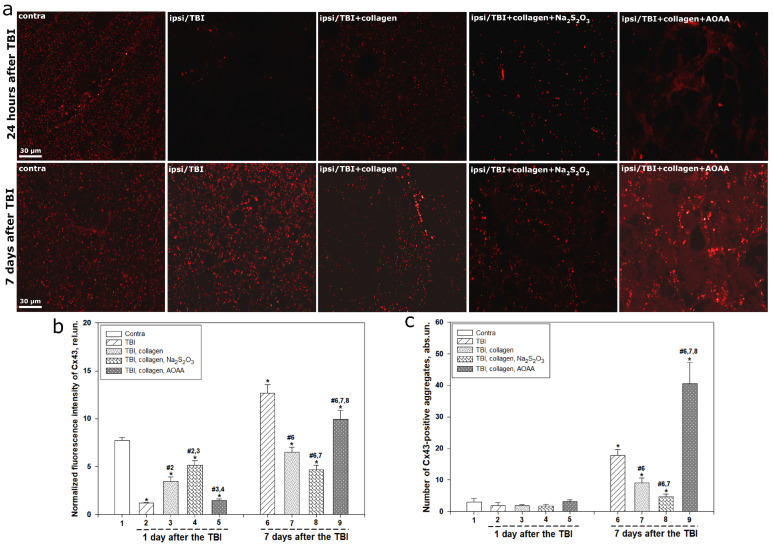
Laser confocal immunofluorescence analysis of Cx43 expression and aggregation in the cerebral cortex following TBI. (**a**) Representative confocal images of Cx43 immunoreactivity in the contralateral hemisphere and ipsilateral cerebral cortex of animals from the experimental groups at 1 and 7 days after TBI: TBI, TBI + collagen matrix, TBI + collagen matrix loaded with Na_2_S_2_O_3_, and TBI + collagen matrix loaded with AOAA. Red fluorescence corresponds to Cx43 immunoreactivity. Scale bar, 30 µm. (**b**) Quantitative analysis of normalized Cx43 fluorescence intensity in the cerebral cortex at 1 and 7 days after TBI. (**c**) Quantitative analysis of the number of Cx43-positive aggregates in the lesion area at 1 and 7 days after TBI. Data are presented as mean ± standard error of the mean (SEM). * *p* < 0.05—statistically significant differences relative to the contralateral hemisphere; # *p* < 0.05—statistically significant differences relative to ipsilateral hemispheres of experimental groups. Significance markers #2, #3, #4, #6, #7, and #8 above bars indicate statistically significant differences between corresponding groups. Data are presented as mean ± SEM (*n* = 6 per group). Statistical analysis was performed using one-way ANOVA followed by the Newman–Keuls post hoc test.

Immunofluorescence analysis revealed pronounced changes in Cx43 expression and distribution in the cerebral cortex following TBI, characterized by alterations in fluorescence intensity and the formation of aggregated Cx43-positive structures within the lesion area (Figure 7a). In the contralateral hemisphere, Cx43 immunoreactivity demonstrated a relatively homogeneous distribution pattern with moderate fluorescence intensity and occasional small Cx43-positive aggregates.

At 1 day after TBI, Cx43 fluorescence intensity in the ipsilateral cortex decreased more than sixfold compared to the contralateral hemisphere (*p* < 0.05; Figure 7b). Implantation of the native collagen matrix was associated with an approximately 2.9-fold increase in Cx43 fluorescence intensity compared to the TBI group (*p* < 0.05). A more pronounced increase in Cx43 immunoreactivity was observed in animals implanted with the collagen matrix loaded with Na_2_S_2_O_3_, where fluorescence intensity increased more than 4.3-fold relative to the TBI group and by approximately 48% compared to the group treated with the native collagen matrix (*p* < 0.05). In contrast, implantation of the AOAA-associated collagen matrix was accompanied by persistently low Cx43 fluorescence intensity, which did not significantly differ from the values observed in the TBI group.

At 7 days after TBI, Cx43 fluorescence intensity markedly increased in the ipsilateral cortex and exceeded the values of the contralateral hemisphere by approximately 60% (*p* < 0.05; Figure 7b). In animals treated with the native collagen matrix, Cx43 fluorescence intensity decreased approximately 2.3-fold compared to the TBI group (*p* < 0.05), whereas implantation of the collagen matrix loaded with Na_2_S_2_O_3_ resulted in an approximately 2.7-fold reduction relative to the TBI group and an approximately 40% decrease relative to the native collagen matrix group (*p* < 0.05). In contrast, animals implanted with the collagen matrix containing AOAA demonstrated significantly higher Cx43 fluorescence intensity compared to both the native collagen matrix group and the Na_2_S_2_O_3_-modified collagen matrix group.

Analysis of Cx43 aggregation revealed no statistically significant differences between experimental groups at 1 day after TBI (Figure 7c), despite the presence of isolated aggregated Cx43-positive structures within the lesion area in all groups. However, at 7 days after injury, differences in the degree of Cx43 aggregation became markedly more pronounced. In the TBI group, a pronounced accumulation of large Cx43-positive aggregates was observed within the perifocal region of the lesion. Implantation of the native collagen matrix was associated with an approximately twofold reduction in the number of aggregated Cx43-positive structures relative to the TBI group, whereas use of the collagen matrix loaded with Na_2_S_2_O_3_ resulted in a marked reduction in Cx43 aggregation—approximately 3.8-fold compared to the TBI group and nearly twofold relative to the native collagen matrix group (*p* < 0.05). In contrast, implantation of the AOAA-associated collagen matrix resulted in the most pronounced Cx43 aggregation, exceeding the values observed in the TBI group by more than 2.2-fold, the native collagen matrix group by approximately 4.4-fold, and the collagen matrix loaded with Na_2_S_2_O_3_ group by more than 8.5-fold (*p* < 0.05; Figure 7c).

### 2.7. Expression and Subcellular Localization of p53 in the Lesion Area After TBI According to Immunofluorescence Analysis

**Figure 8:**
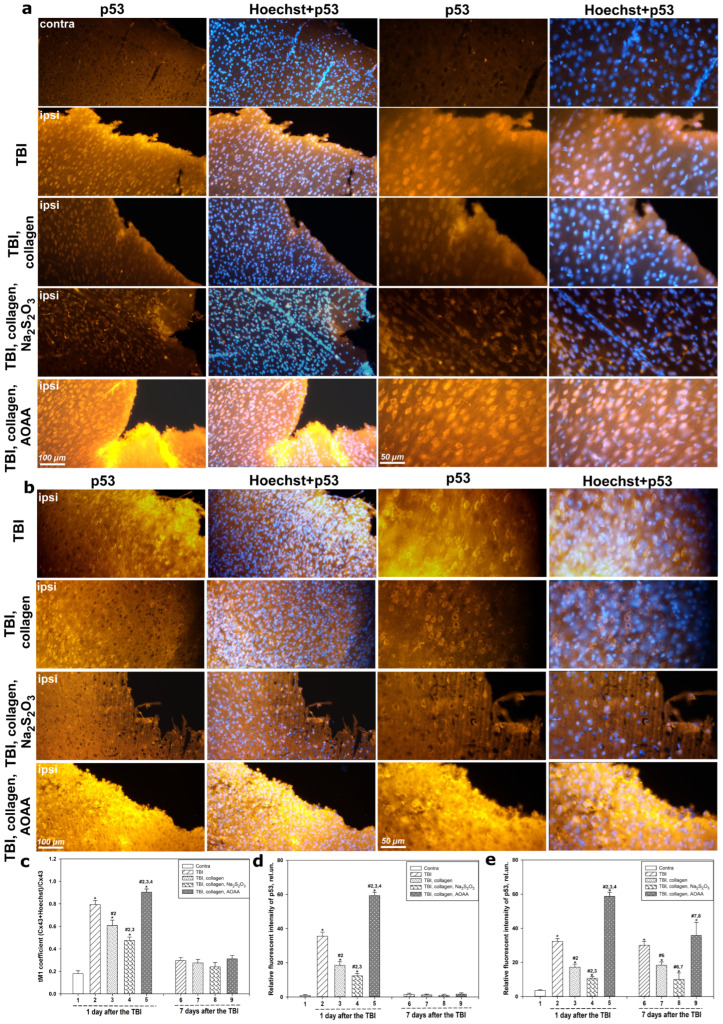
Immunofluorescence analysis of p53 expression and subcellular localization in the cerebral cortex after TBI. (**a**) Immunofluorescence microscopy 1 day after TBI, showing p53 expression (yellow fluorescence) and Hoechst 33342 staining (blue fluorescence); merged signals are presented in the p53 + Hoechst panel; scale bars: 100 μm and 50 μm. (**b**) Immunofluorescence microscopy 7 days after TBI; fluorescent signal designations and scale bars are the same as in panel (**a**). (**c**) M1 colocalization coefficient between p53 and Hoechst signals; (**d**) mean fluorescence intensity of p53 in the nuclear region; (**e**) mean fluorescence intensity of p53 in the cytoplasmic region. Groups: Contra—contralateral cortex; TBI—ipsilateral cortex 1 day after TBI; TBI + collagen—TBI with collagen matrix implantation; TBI + collagen + Na_2_S_2_O_3_—TBI with collagen matrix loaded with Na_2_S_2_O_3_; TBI + collagen + AOAA—TBI with collagen matrix under CBS inhibition (AOAA). For panels (**c**–**e**), the first five bars correspond to day 1 after TBI, and the subsequent four bars correspond to day 7 after TBI. * *p* < 0.05—statistically significant differences relative to the contralateral hemisphere; # *p* < 0.05—statistically significant differences relative to ipsilateral hemispheres of experimental groups. Significance markers #2, #3, #4, #6, #7, and #8 above bars indicate statistically significant differences between corresponding groups. Data are presented as mean ± SEM (*n* = 6 per group). Statistical analysis was performed using one-way ANOVA followed by the Newman–Keuls post hoc test.

Immunofluorescence microscopy combined with quantitative analysis of fluorescence intensity and the M1 colocalization coefficient allowed characterization of the phase-dependent dynamics of p53 expression and subcellular distribution in the cerebral cortex following TBI, as well as evaluation of the effects of pharmacological modulation of H_2_S levels on the post-traumatic cellular response. The analysis revealed pronounced changes in both nuclear and cytoplasmic localization of p53 during the acute phase of TBI (Figure 8a), followed by nuclear–cytoplasmic translocation at later stages after injury (Figure 8b).

In the contralateral hemisphere, p53 levels remained consistently low and were characterized by weak immunoreactivity in both the nuclear and cytoplasmic compartments, reflecting the basal expression level of this protein under physiological conditions. The M1 colocalization coefficient showed low values, corresponding to limited nuclear localization of p53 in intact tissue (Figure 8c). The signal was predominantly weak and diffuse, without pronounced intracellular accumulation (Figure 8a).

At 24 h after TBI, a significant increase in p53 expression was observed in the ipsilateral cortex, accompanied by pronounced accumulation of p53 in both nuclear and cytoplasmic regions of the cells. The M1 coefficient increased more than fourfold compared to the contralateral hemisphere (*p* < 0.05), indicating a marked elevation of nuclear p53 levels (Figure 8c). These changes were confirmed by an increase in p53 fluorescence intensity in the nuclear region (*p* < 0.05; Figure 8d). At the same time, a substantial increase in cytoplasmic p53 was observed, with fluorescence intensity rising more than ninefold compared to the contralateral cortex (*p* < 0.05; Figure 8e).

Application of the collagen matrix was associated with a partial reduction in nuclear p53 localization compared to the TBI group, as evidenced by a 23% decrease in the M1 coefficient (*p* < 0.05; Figure 8c) and a 48% reduction in nuclear fluorescence intensity (*p* < 0.05; Figure 8d). The most pronounced decrease in p53 levels was observed in the group treated with collagen matrix loaded with Na_2_S_2_O_3_, where the M1 coefficient decreased by 40% relative to the TBI group (*p* < 0.05; Figure 8c), and nuclear fluorescence intensity was reduced nearly threefold (*p* < 0.05; Figure 8d). Importantly, animals implanted with the collagen matrix loaded with Na_2_S_2_O_3_ also demonstrated significantly lower M1 colocalization coefficients (Figure 8c) and reduced nuclear p53 fluorescence intensity (Figure 8d) compared to animals treated with the native collagen matrix alone (*p* < 0.05), indicating a more pronounced suppression of nuclear p53 accumulation following pharmacological modification of the scaffold. Cytoplasmic p53 intensity in this group was also significantly reduced (*p* < 0.05; Figure 8e). Moreover, cytoplasmic p53 fluorescence intensity in animals implanted with the collagen matrix loaded with Na_2_S_2_O_3_ was significantly lower compared to animals treated with the native collagen matrix alone (*p* < 0.05), suggesting additional attenuation of p53-associated stress responses under conditions of local Na_2_S_2_O_3_ delivery (Figure 8e).

In contrast, inhibition of CBS using AOAA resulted in a marked increase in p53 expression. The M1 coefficient increased by 14% compared to the TBI group (*p* < 0.05; Figure 8c). In addition, animals implanted with the AOAA-loaded collagen matrix demonstrated significantly higher M1 colocalization coefficients compared both to animals treated with the native collagen matrix and to animals implanted with the collagen matrix loaded with Na_2_S_2_O_3_ (*p* < 0.05, Figure 8c). Fluorescence intensity of p53 in both the nucleus (Figure 8d) and cytoplasm (Figure 8e) reached the highest levels among all experimental groups (*p* < 0.05). Nuclear (Figure 8d) and cytoplasmic (Figure 8e) p53 fluorescence intensities in the AOAA-treated group were also significantly increased relative to both the native collagen matrix group and the collagen matrix loaded with Na_2_S_2_O_3_ (*p* < 0.05), indicating enhanced activation of p53-associated stress and apoptotic signaling under inhibition of endogenous H_2_S synthesis.

At 7 days after TBI, a pronounced shift in the subcellular distribution of p53 was observed. The M1 coefficient values significantly decreased compared to the 24 h group and did not differ between experimental groups (Figure 7c). Nuclear p53 fluorescence intensity in all groups approached the levels observed in the contralateral hemisphere (Figure 8d). However, cytoplasmic p53 levels remained elevated in all TBI groups, with fluorescence intensity significantly exceeding that of the contralateral cortex (*p* < 0.05). Statistically significant differences between experimental groups persisted (*p* < 0.05; Figure 8e). In particular, animals implanted with the collagen matrix loaded with Na_2_S_2_O_3_ demonstrated significantly lower cytoplasmic p53 fluorescence intensity compared to animals treated with the native collagen matrix alone, whereas AOAA treatment resulted in significantly higher cytoplasmic fluorescence levels relative to both the native collagen matrix group and the Na_2_S_2_O_3_-loaded collagen matrix group (*p* < 0.05) (Figure 8e). Additionally, an increase in diffuse p53 signal in the extracellular space within the lesion area was observed, manifested as elevated background fluorescence of the neuropil (Figure 8b).

### 2.8. Ultrastructural Analysis of Pathological Changes in Nervous Tissue Following TBI

**Figure 9:**
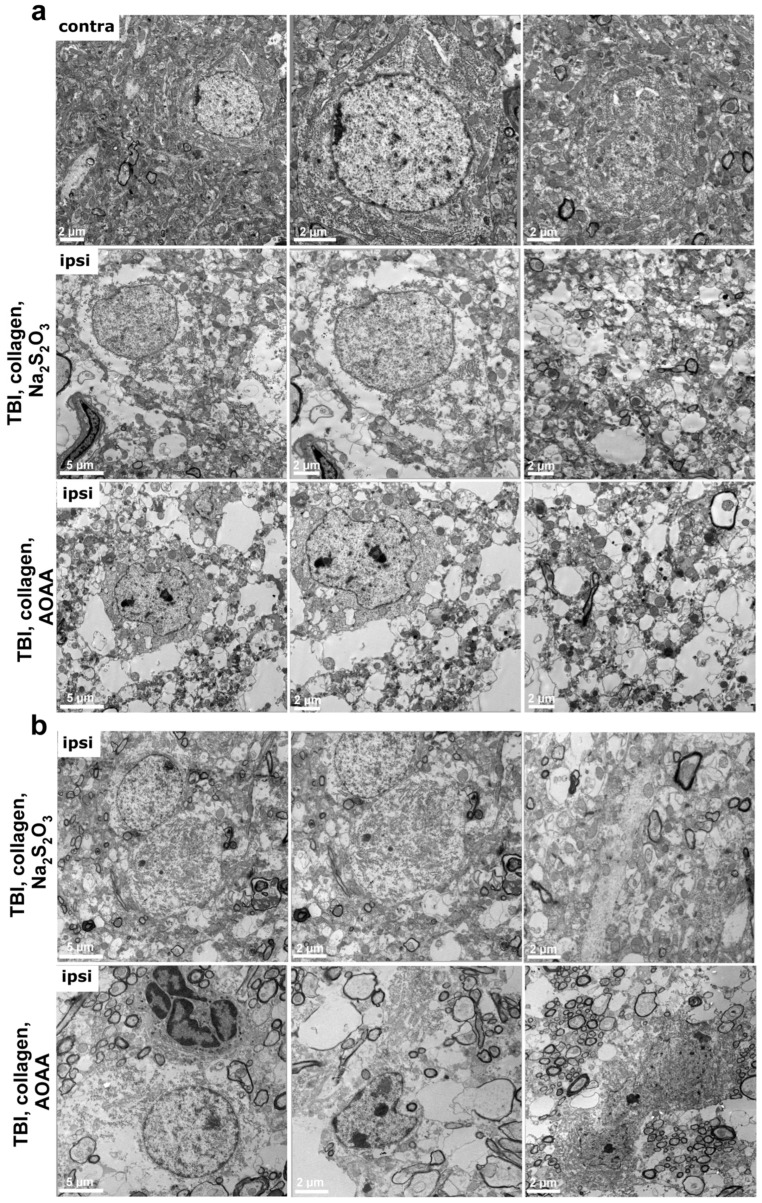
Ultrastructural changes in the cerebral cortex of mice assessed by transmission electron microscopy at 1 and 7 days after severe TBI with collagen matrix implantation. (**a**) 1 day after TBI; (**b**) 7 days after TBI. The images show ultrastructural changes in the contralateral (intact) and ipsilateral (injured) hemispheres in the following experimental groups: TBI + collagen matrix loaded with Na_2_S_2_O_3_ and TBI + collagen matrix with AOAA.

Ultrastructural analysis demonstrated that TBI induces significant alterations in the architecture of nervous tissue; however, in groups with implantation of a collagen matrix containing Na_2_S_2_O_3_, a less pronounced degree of damage was observed. In the intact contralateral hemisphere, neurons preserved their normal morphology, with clearly defined plasma membranes and evenly distributed chromatin. The cytoplasm contained numerous ribosomes, mitochondria were observed in neuronal somata and their processes, and both the rough endoplasmic reticulum and the Golgi apparatus were well distinguished. Dendrites and axons also retained normal structure without signs of edema or vacuolization, and the myelin sheath of nerve fibers remained dense and homogeneous (Figure 9).

At 24 h after TBI, more pronounced alterations were observed in the group with implantation of the collagen matrix containing AOAA, where neuronal plasma membranes were markedly thinned or completely disrupted. The nuclear envelope underwent degradation, and the cytoplasm was disorganized with fragmentation of intracellular compartments. The number of mitochondria was significantly reduced, and the remaining organelles exhibited clear signs of severe damage. In addition, the myelin sheath of axons showed multiple ruptures and splitting. Some neurons were completely destroyed. In contrast, in the group with implantation of the collagen matrix loaded with Na_2_S_2_O_3_, fewer destructive changes were observed: mitochondria and membranes remained more intact, as did myelinated fibers and dendrites compared to the AOAA group (Figure 9a).

At 7 days after TBI, tissue alterations in the group with implantation of the collagen matrix containing Na_2_S_2_O_3_ were less pronounced than in the AOAA group. Although destructive changes continued to progress, nervous tissue in the Na_2_S_2_O_3_-treated group retained a higher degree of structural organization, and cellular elements were not completely destroyed. Microglial cells engaged in phagocytosis of damaged regions were less numerous, and the tissue exhibited fewer signs of irreversible damage. In contrast, the AOAA group showed more severe tissue alterations, with progression of phagocytic activation and the presence of extensive damage (Figure 9b).

### 2.9. In Silico Study

#### 2.9.1. Conformational Dynamics and Binding Features of Sulfur-Containing Ligands with Cx43 Under Physiological and Ischemic Conditions

**Figure 10:**
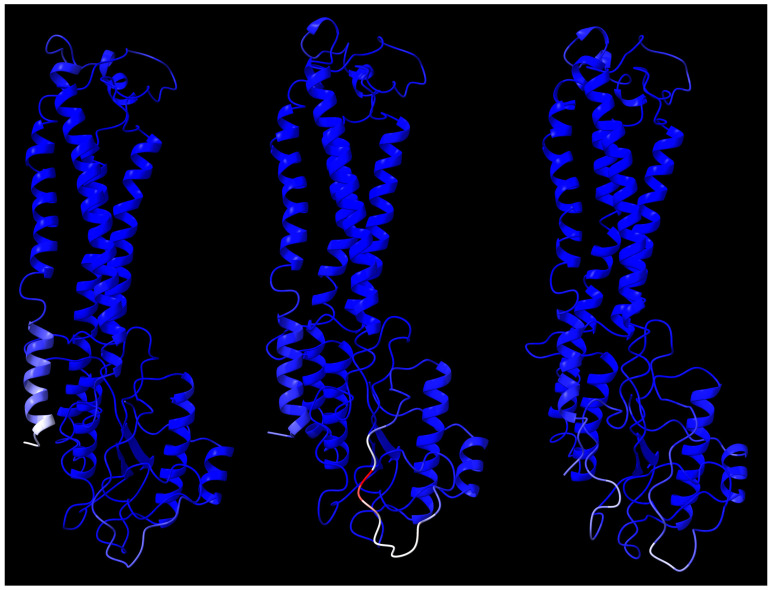
Heat maps of fluctuations of Cx43 amino acid residues under different simulation conditions. Three systems are presented: the physiological model (**Left**), the protonated model at pH 6.5 (**Center**), and the ischemic model (low pH in the presence of Ca^2+^ ions, **Right**). Blue indicates regions with the lowest mobility, whereas red indicates regions with the highest mobility during molecular dynamics simulations.

Analysis of the conformational dynamics of Cx43 (Figure 10) revealed several characteristic patterns. Under physiological conditions, the cytosolic domain (amino acid residues 1–18), which functions as the “entry gate” of the hemichannel, exhibited increased conformational mobility compared to protonated forms of the protein, including the system containing Ca^2+^ ions. The reduction in mobility of this region under acidic conditions may reflect alterations in channel regulatory properties. We suppose that this mechanism causes interneuronal communication during ischemia.

**Figure 11:**
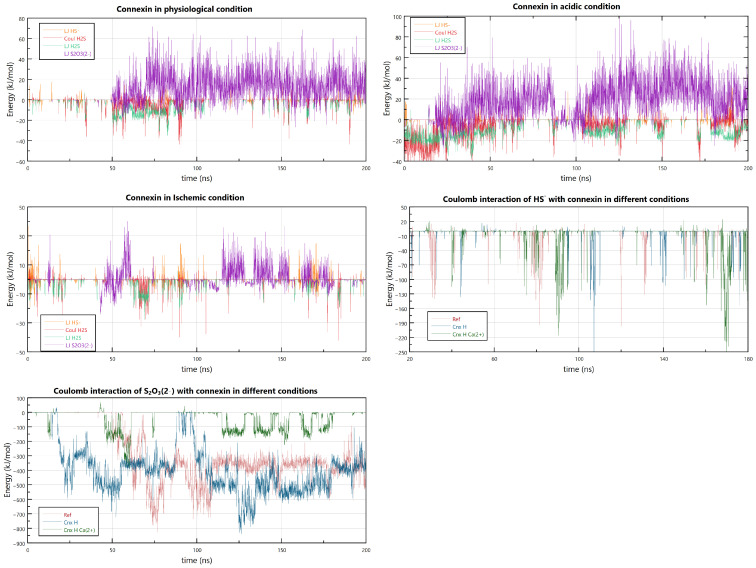
Time-dependent dynamics of Coulomb (Coul) and van der Waals (LJ) interactions between Cx43 and ligands HS^−^, H_2_S, and S_2_O_3_^2−^ under physiological, acidic, and ischemic conditions. The graphs are grouped by energy ranges for improved visualization.

In an acidic environment, an increase in mobility of the cytosolic domain fragment comprising residues 331–344 was also observed (red–white region in the central structure, Figure 10). The addition of Ca^2+^ ions partially compensated for this effect, leading to stabilization of the conformation of this domain. The results of interaction energy analysis are presented in Table 1 and Figure 11.

Analysis of the energetic parameters indicates that HS^−^ and H_2_S molecules do not form stable noncovalent complexes with Cx43 within the simulated time interval. Nevertheless, their potential regulatory role cannot be excluded, as one of the key mechanisms of H_2_S signaling is covalent modification of cysteine thiol groups via S-sulfhydration [19]. Modeling of such covalent modifications requires specialized parameterization and is beyond the scope of the present study.

**Figure 12:**
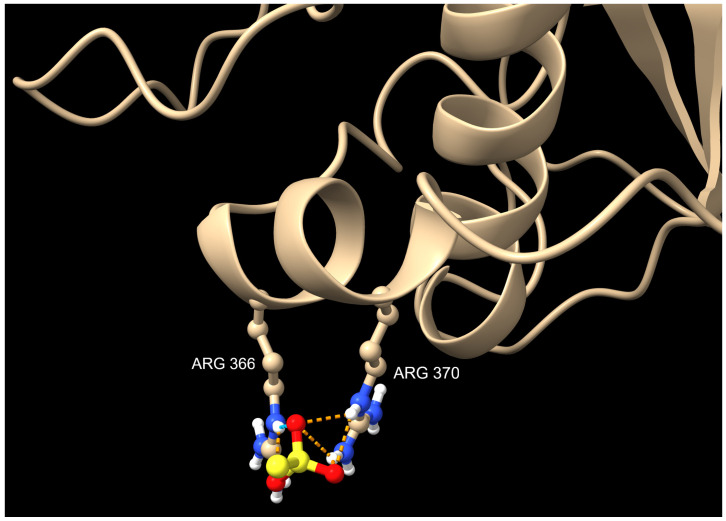
Cytosolic domain of Cx43 involved in S_2_O_3_^2−^ binding.

The most pronounced interaction was observed for the S_2_O_3_^2−^ anion. It forms stable electrostatic contacts with arginine residues at positions 366 and 370 (Figure 12). Significant negative values of Coulomb energy (Table 1, Figure 11) indicate the stability of this interaction. It can be assumed that S_2_O_3_^2−^ may act as an electrostatic “adapter,” partially compensating for the positive charge of the domain and facilitating the formation of protein–protein complexes involving other positively charged regions. Under ischemic conditions, the presence of Ca^2+^ ions weakens the formation of this complex, likely due to competition for binding with negatively charged S_2_O_3_^2−^ ions. Nonetheless, the MDS results exhibit significant system instability (indicated by high Total Energy Drift and RMSD values) and should be considered hypothesis-generating observations only. Additional in silico studies are required to better explain the experimental biological results.

#### 2.9.2. Conformational Stability of p53 and Features of Zn^2+^ Coordination and Sulfur-Containing Ligand Interactions Under Different Environmental Conditions

**Figure 13:**
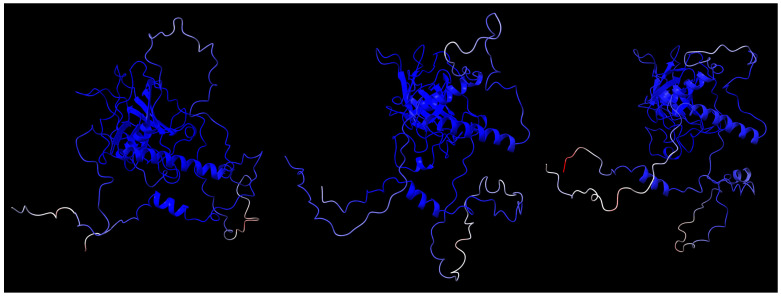
Heat maps of fluctuations of p53 amino acid residues under different conditions. Models are presented for physiological conditions (reference, **left**), acidic conditions (**center**), and ischemic conditions (low pH with Ca^2+^ ions, **right**). Blue indicates regions with the lowest mobility during molecular dynamics simulations, whereas red indicates regions with the highest mobility across all three models.

Conformational analysis of the p53 protein (Figure 13) demonstrates that protonation and the presence of Ca^2+^ ions lead to increased mobility of intrinsically disordered domains of the protein. Increased lability of these regions may affect the ability of p53 to specifically bind DNA and, consequently, its regulatory functions.

Particular attention was given to the Zn^2+^ coordination domain, which plays a key role in maintaining the spatial structure of p53. In the models, the Zn^2+^ ion was initially positioned within the corresponding coordination site, followed by energy minimization and system equilibration. However, under all examined conditions, gradual dissociation of Zn^2+^ from the coordination site was observed, indicating insufficient stability of the complex in the absence of additional stabilizing factors.

**Figure 14:**
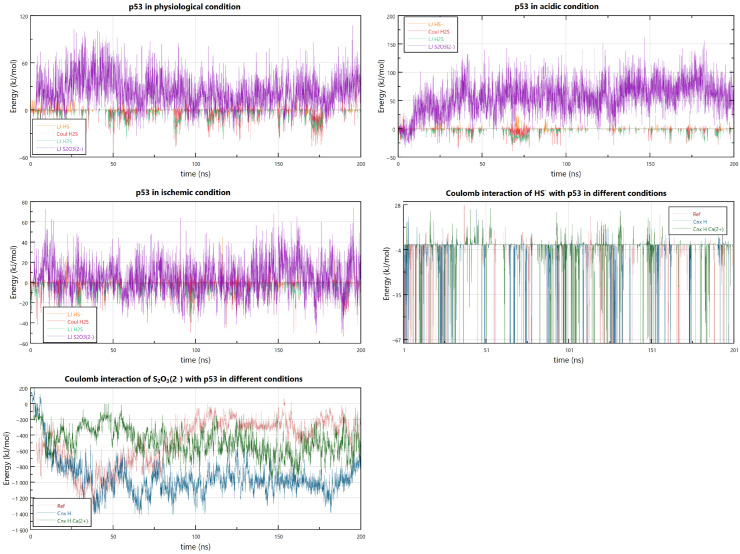
Time-dependent dynamics of Coulomb (Coul) and van der Waals (LJ) interactions between p53 and HS^−^, H_2_S, and S_2_O_3_^2−^ under physiological, acidic, and ischemic conditions. The graphs are grouped according to energy ranges for clarity.

In protonated forms of p53, not only histidine residues but also cysteine residues (positions 176, 238, and 242) become protonated, further reducing the stability of the zinc coordination center. Therefore, decreased intracellular pH in neurons may potentially lead to destabilization of the active form of p53 and impairment of its functional activity. In addition, negatively charged ligands such as HS^−^ and S_2_O_3_^2−^ are capable of coordinating around Zn^2+^, competing for binding and further reducing the likelihood of forming a functionally active complex. The results of interaction energy analysis are presented in Table 2 and Figure 14.

**Figure 15:**
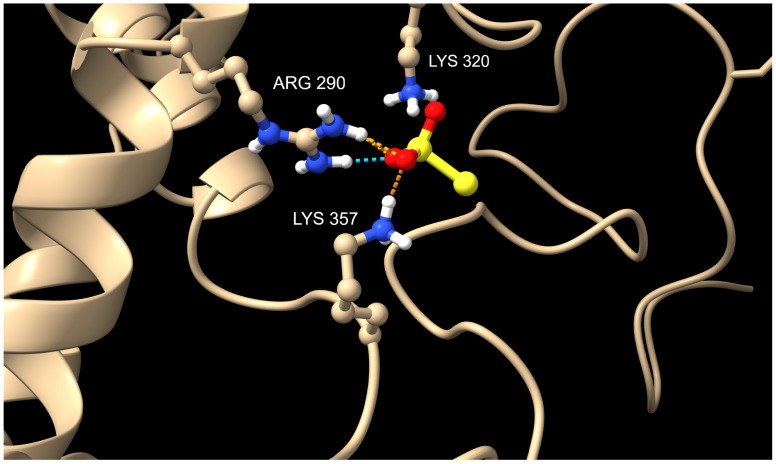
Structural organization of the p53 domain (residues 290, 320, 357) involved in S_2_O_3_^2−^ binding. Dashed lines indicate hydrogen bonds; the blue line denotes a hydrogen bond geometry close to the canonical configuration.

In contrast to Cx43, the protonated form of p53 demonstrates an increased propensity for binding S_2_O_3_^2−^, as evidenced by more pronounced Coulomb interaction energies. These findings are consistent with the assumption that divalent anions tend to shield positively charged centers of the protein. Under ischemic conditions, Ca^2+^ ions compete with S_2_O_3_^2−^ for binding to positively charged domains of p53, leading to a weakening of these interactions (Figure 14). Figure 15 shows the structural organization of the domain (aa 290, 320, 357) in which effective binding of the S_2_O_3_^2−^ molecule occurs.

The results suggest a potential role of S_2_O_3_^2−^ in modulating the structural dynamics of p53 and its interactions with binding partners; however, additional computational and experimental studies are required to reveal this mechanism. Additional molecular dynamics data supporting the observed structural changes are provided in the Supplementary Materials.

## 3. Discussion

The present study provides the first comprehensive evaluation of the potential of a collagen matrix derived from the jellyfish *R. pulmo* and modified with Na_2_S_2_O_3_ as a potential source of H_2_S-associated effects, as a bioengineered construct for neuroprotection and tissue engineering in a mouse model of severe TBI. The obtained data demonstrate that combining the structural advantages of marine type I collagen, including high porosity, biocompatibility, and hydrophilicity, with pharmacological modulation of the H_2_S gasotransmitter pathway may contribute to modulation of several key aspects of the post-traumatic process: mechanical defect filling, suppression of p53-dependent apoptosis, partial preservation of Cx43 expression, and attenuation of reactive astrogliosis. This multilevel approach opens new prospects for the development of fundamentally novel biodegradable implants capable not only of replacing lost tissue but also of potentially influencing molecular mechanisms involved in secondary brain injury—a problem that remains unresolved in clinical neurotraumatology.

The electrophoretic profile of collagen from *R. pulmo* jellyfish revealed submolecular fractions in the range of approximately 95–100 and 105 kDa, as well as a dimeric fraction in the high molecular weight region, consistent with literature data for type I collagen from jellyfish [20]. For *R. pulmo* and other jellyfish species, the presence of α1- and α2-chains in the range of approximately 92–125 kDa and β-chains above 150 kDa has previously been reported, which is in agreement with our findings [21,22]. The presence of β-dimers indicates preservation of interchain interactions, whereas the characteristic diffuse band pattern reflects the electrophoretic behavior of fibrillar collagen and does not contradict its typical structure [23]. Thus, the observed electrophoretic pattern confirms that the isolated protein belongs to type I collagen and corresponds to previously described jellyfish collagen in terms of its submolecular characteristics.

Fluorescence analysis of the collagen matrix structure further demonstrated that collagen sponges derived from *R. pulmo* form a pronounced three-dimensional porous architecture with a system of interconnected pores uniformly distributed throughout the volume. This feature is critically important for their application as tissue engineering scaffolds and is consistent with previous reports highlighting the potential of jellyfish-derived collagen for biomedical use. The use of fluorescent dyes such as Hoechst 33342, Propidium iodide, and SYTOX Green in combination with fluorescence microscopy enabled visualization of pore spaces and septa that are not detectable under transmitted light, underscoring the methodological efficiency of this approach compared to more labor-intensive techniques such as transmission electron microscopy. Although these dyes are traditionally used as nuclear markers, in this study they demonstrated high informativeness for analyzing collagen matrix structure. This is likely due to their ability to penetrate water-filled pores and contrast the internal architecture of the sponge, making them a convenient tool for assessing porous biomaterials. The high swelling capacity of the sponges and their pronounced hydrophilic properties, previously demonstrated in swelling kinetics studies [13], correlate well with the observed porous structure and indicate significant potential of the material as an absorbent and water-retaining biomaterial for biomedical applications, particularly for the treatment of severe TBI.

The presented data on implantation of the collagen matrix into the TBI site show that the severe TBI model using a freely falling weight reproducibly generates a large cortical defect with a characteristic pattern of acute vascular injury. A key finding is the behavior of the implanted collagen matrix, which tightly fills the defect, maintains a stable position, and provides a three-dimensional scaffold for granulation tissue formation. This is associated with a reduction in cystic cavity formation and thinning of the adjacent cortex compared to animals without implantation. Good tolerance of the procedure by most animals, despite isolated mortality cases, indicates an acceptable level of safety of the material and implantation method. These findings are consistent with numerous previous studies demonstrating the safety and efficacy of collagen scaffolds for reconstruction of post-traumatic brain defects [24,25,26]. Moreover, marine collagen derived from jellyfish exhibits comparable or superior properties to mammalian collagen in terms of reduced immunogenicity and enhanced regenerative potential [27].

Although the Kaplan–Meier analysis did not reveal statistically significant differences between the experimental groups, animals treated with AOAA demonstrated a tendency toward reduced survival, reaching 75%, whereas survival in the groups implanted with the native collagen matrix and the Na_2_S_2_O_3_-modified matrix remained at 100%. Previous studies have shown that inhibition of endogenous H_2_S synthesis may negatively affect post-traumatic outcomes [28]. At the same time, the relatively small sample size used in the present study may have limited the statistical power of the analysis and reduced sensitivity for detecting moderate intergroup differences. Therefore, the obtained results should be interpreted with caution, and further validation in studies involving larger experimental cohorts is required.

In our recent work, collagen isolated from *R. pulmo* was also shown to possess high biocompatibility, promote cell adhesion, migration, and proliferation, and support extracellular matrix formation [13]. Our results are consistent with these findings and further demonstrate that the three-dimensional porous structure of the collagen matrix provides a favorable environment for granulation tissue formation and may contribute to limiting secondary brain injury following TBI.

Further histological examination revealed areas of pronounced glial reaction and forming scar tissue at the periphery of the defect, which represents a typical morphological pattern of central nervous system injury following focal trauma and reflects the development of reactive astrogliosis aimed at limiting the lesion area and restoring the barrier properties of the tissue [29,30]. In animals without matrix implantation, the post-traumatic lesion over time demonstrated a tendency toward the formation of a pronounced cystic cavity with thinning of the adjacent cortical layers, which is a characteristic consequence of necrosis and loss of neuroglial interactions in the primary injury zone [31], whereas in the group with the collagen scaffold, the cavity was largely filled with the implant and granulation tissue. This dynamic highlights not only the structural role of the collagen matrix as a temporary scaffold but also its ability to guide reparative processes, preventing progressive atrophy, cell death, and cystic degeneration of brain tissue.

Quantitative histological analysis confirms the observed morphological findings, demonstrating a pronounced phase-dependent dynamic of post-traumatic morphological changes in nervous tissue following TBI. As early as 1 day after TBI, a significant increase in the proportion of pathologically altered cells was observed, characterized by signs of pyknosis, karyolysis, and cytoplasmic vacuolization, reflecting activation of necrotic and apoptotic cell death processes in the lesion area. At the same time, marked rarefaction of the neuropil and the appearance of foci of inflammatory infiltration were observed, indicating early activation of the neuroinflammatory response. Application of the collagen matrix was associated with a significant reduction in the proportion of pathologically altered cells. The most pronounced neuroprotective effect was observed when using a collagen matrix loaded with Na_2_S_2_O_3_, which is consistent with the known antioxidant and anti-inflammatory properties of H_2_S donors [32]. It is known that Na_2_S_2_O_3_ has been proposed as a compound capable of exerting H_2_S-associated biological effects under conditions of hypoxia, acidosis, and oxidative stress characteristic of the injury site [32]. In our previous study, we also demonstrated the neuroprotective potential of Na_2_S_2_O_3_ in TBI treatment, where its use contributed to a reduction in the severity of nervous tissue damage [18]. It should be noted that Na_2_S_2_O_3_ has demonstrated efficacy in the treatment of cerebral ischemia–reperfusion injury [33,34], Alzheimer’s disease [35], and neuroinflammation induced by LPS and IFNγ [36]. In contrast, the use of a collagen matrix associated with AOAA—which inhibits CBS, the key enzyme responsible for H_2_S synthesis in nervous tissue [37]—resulted in opposite effects. However, it should be noted that the precise molecular mechanisms underlying the biological effects of Na_2_S_2_O_3_ remain incompletely understood. Although Na_2_S_2_O_3_ is widely considered a potential source of H_2_S-associated signaling under pathological conditions, it is still unclear whether its effects are mediated predominantly through direct H_2_S release, sulfur transfer reactions, or intrinsic biological properties of thiosulfate itself. Previous studies have suggested that thiosulfate may exert independent cytoprotective, antioxidant, and anti-inflammatory effects that are not exclusively dependent on H_2_S generation [10]. Therefore, the neuroprotective effects observed in the present study cannot be unequivocally attributed solely to H_2_S release and may also involve direct biological actions of thiosulfate.

At 7 days after injury, morphological changes became more pronounced, and an increase in cell density in the perifocal area was observed, likely due to the development of reactive gliosis, which is a characteristic stage of the reparative process following CNS injury [29]. Despite the persistence of an elevated proportion of pathologically altered cells compared to the contralateral hemisphere, application of the collagen matrix contributed to a reduction in the severity of degenerative changes in brain tissue, whereas the use of AOAA was associated with exacerbation of damage, as evidenced by a higher level of cellular destruction. These findings indicate that the collagen matrix may serve as a carrier for Na_2_S_2_O_3_, potentially contributing to local exposure of the lesion area to sulfur-containing compounds and to the observed neuroprotective effects, which may be associated both with Na_2_S_2_O_3_-related signaling and with the structural support provided by the collagen carrier.

Analysis of the area of inflammatory infiltration revealed a marked increase in this parameter in the early post-traumatic period, reflecting activation of the innate immune response, disruption of blood–brain barrier permeability, and migration of inflammatory cells into the lesion area. The use of the collagen matrix led to a reduction in the infiltration area compared to the TBI group, which may indicate attenuation of secondary inflammatory damage in brain tissue. The most pronounced effect was again observed with collagen loaded with Na_2_S_2_O_3_, whereas inhibition of H_2_S synthesis using AOAA was associated with an enhanced inflammatory response. At 7 days after injury, the infiltration area remained elevated relative to intact tissue; however, differences between experimental groups were no longer significant, which may indicate a transition of the inflammatory process from the acute phase to the tissue remodeling stage.

In addition, ultrastructural analysis confirmed that TBI is accompanied by pronounced neuronal destruction and disruption of neuropil organization; however, the use of a collagen matrix loaded with Na_2_S_2_O_3_ contributed to partial preservation of cellular architecture. Greater preservation of plasma membranes, mitochondria, and myelinated fibers, as well as less pronounced degenerative changes compared to the AOAA group, indicate a neuroprotective effect of H_2_S. This effect is likely associated with reduced mitochondrial dysfunction, limitation of oxidative stress, and stabilization of cellular membranes, which collectively contribute to slowing the progression of secondary damage in nervous tissue after TBI.

Another potential mechanism associated with the observed neuroprotective effects may involve the H_2_S-dependent modulation of the expression and subcellular localization of the tumor suppressor protein p53—one of the central regulators of the cellular response to damage, known as the “guardian of the genome,” which controls apoptosis, cell cycle progression, DNA repair, and mitochondrial dysfunction [38]. In the present study, it was shown that in the acute phase, 24 h after TBI, pronounced nuclear and cytoplasmic accumulation of p53 was observed in the injured area. This pattern is consistent with post-traumatic apoptosis-associated changes and secondary injury: nuclear p53 activates transcription of pro-apoptotic genes, whereas cytoplasmic p53 directly interacts with mitochondrial Bcl-2 family proteins, promoting cytochrome c release, activation of the caspase cascade, and irreversible neuronal death [39]. These findings are consistent with our recent study, in which increased p53 expression in both nuclear and cytoplasmic compartments of neurons and glial cells was observed 24 h after a similar cortical injury [18]. In addition, in our previous studies using another TBI model, a similar pattern was observed at early time points after injury [16]. Other studies also indicate a rapid increase in p53 levels as early as 15 min after TBI, with sustained elevation for at least the first 24 h [40].

At 7 days after injury, p53 levels in the nuclear fraction significantly decreased and approached values characteristic of intact tissue, whereas cytoplasmic localization of the protein remained elevated. This may indicate a shift in p53 toward predominantly transcription-independent mechanisms of regulating the cellular response to injury, including involvement in mitochondria-mediated apoptotic pathways and stress-induced signaling cascades [41]. Notably, a similar nuclear-to-cytoplasmic redistribution of p53, characterized by decreased nuclear localization and increased cytoplasmic levels, was previously observed by us in dorsal root ganglia following axotomy [42], suggesting common p53-dependent signaling responses to traumatic injury in both the peripheral and central nervous systems.

Implantation of a collagen matrix loaded with Na_2_S_2_O_3_ resulted in the most pronounced reduction in both nuclear and cytoplasmic p53 levels, whereas inhibition of CBS using AOAA, in contrast, enhanced p53 accumulation. The neuroprotective effect of Na_2_S_2_O_3_ may be mediated through several interconnected mechanisms, including antioxidant activity, stabilization of mitochondrial membranes, suppression of pro-inflammatory glial activation [33,34,35,36], and direct or indirect inhibition of p53-dependent apoptotic pathways [18]. For example, H_2_S may participate in regulation of the p53/MDM2 signaling pathway, which represents one of the key mechanisms controlling the cellular response to stress and injury. MDM2 is known to be the principal E3 ubiquitin ligase of p53, regulating its ubiquitination, proteasomal degradation, and subcellular localization [43]. H_2_S is capable of modulating p53 activity both indirectly—through effects on the MDM2/p53 signaling pathway and ubiquitination processes [44]—and directly via redox-dependent post-translational modifications of the protein, including S-sulfhydration of cysteine residues [45,46]. Regulation of the p53/MDM2 system following neural tissue injury is considered one of the key mechanisms determining the balance between survival and death of neurons and glial cells under conditions of oxidative stress, neuroinflammation, and mitochondrial dysfunction [47,48]. In this regard, it cannot be excluded that the reduction in p53 accumulation observed in the present study following application of the collagen matrix modified with Na_2_S_2_O_3_ may be partially associated with alterations in MDM2-dependent mechanisms regulating p53 activity.

The data obtained in this study are consistent with these observations and extend current understanding by demonstrating that local administration of Na_2_S_2_O_3_ using a collagen matrix derived from *R. pulmo* was associated with reduced p53 accumulation in both nuclear and cytoplasmic compartments of neurons and glial cells following TBI. In contrast, pharmacological inhibition of CBS using AOAA exacerbates H_2_S deficiency and was associated with increased apoptosis-related changes and morphological damage, which is consistent with evidence that inhibition of endogenous H_2_S synthesis aggravates secondary injury after TBI. It is noteworthy that in one study, the neuroprotective effect of an injectable SF hydrogel with sustained H_2_S delivery, which reduces neuronal pyroptosis in severe intracerebral hemorrhage, was also abolished by AOAA [49].

It should be noted that excessive inhibition of apoptotic signaling pathways may theoretically contribute to a shift in cell death toward alternative forms of regulated cell death, including necroptotic mechanisms [50], which are associated with pronounced inflammatory responses and expansion of secondary injury progression in nervous tissue following TBI [51]. At present, different forms of cell death are considered a complex interconnected network of molecular processes capable of dynamic reorganization in response to pharmacological interventions and changes in the microenvironment of damaged tissue [52]. In the present study, morphological and ultrastructural analyses did not reveal signs of aggravated pathological alterations in nervous tissue following application of the collagen matrix modified with Na_2_S_2_O_3_. On the contrary, reduced severity of destructive changes in brain tissue, decreased inflammatory infiltration, and better preservation of cellular ultrastructure were observed.

Characteristic biphasic changes in the expression of Cx43—the principal gap junction protein of astrocytes, playing a critical role in intercellular communication, glutamate homeostasis, propagation of Ca^2+^ waves, and limitation of secondary injury—were also observed. In the acute phase, already at 24 h after TBI, there was an almost complete loss of Cx43 immunoreactivity in the context of reactive astrogliosis. Quantitative analysis additionally confirmed a more than sixfold reduction in Cx43 fluorescence intensity in the ipsilateral cortex compared to the contralateral hemisphere during the acute post-traumatic period. Implantation of the native collagen matrix partially restored Cx43 expression, whereas the collagen matrix loaded with Na_2_S_2_O_3_ demonstrated the most pronounced preservation of Cx43 immunoreactivity. In contrast, inhibition of endogenous H_2_S synthesis using AOAA was associated with persistently low Cx43 fluorescence intensity, comparable to the untreated TBI group, indicating aggravation of early post-traumatic impairment of astroglial gap junction organization. By 7 days after injury, Cx43 levels markedly increased, accompanied by the formation of large aggregates. Quantitative analysis additionally demonstrated that delayed post-traumatic hyperexpression of Cx43 was associated with pronounced aggregation of Cx43-positive structures within the perifocal region. At this stage, implantation of the collagen matrix loaded with Na_2_S_2_O_3_ was associated with a marked reduction in both Cx43 fluorescence intensity and the number of aggregated Cx43-positive structures compared to the TBI group and the group treated with the native collagen matrix. Animals treated with AOAA demonstrated the most pronounced aggregation of Cx43 despite fluorescence intensity values remaining slightly lower than those observed in the untreated TBI group. These findings may indicate that inhibition of endogenous H_2_S synthesis promotes pathological redistribution and clustering of Cx43 into large non-functional aggregates rather than uniform membrane-associated expression of the protein. This dynamic is likely associated both with excessive synthesis of Cx43 and impairment of its intracellular degradation mechanisms. These results are fully consistent with our previous studies, which demonstrated a similar biphasic pattern of Cx43 changes [14,15], as well as with numerous reports from other authors highlighting the complex and phase-dependent regulation of Cx43 following TBI [53,54,55,56]. It should be noted that loss of functional activity of Cx43 has been associated with disruption of intercellular communication via gap junctions [12]. This, in turn, triggers a cascade of neurotoxic processes, including a marked increase in the inflammatory response, significant reduction in the resistance of neurons and astrocytes to oxidative and metabolic stress, and impaired regeneration of damaged cells. In addition, dysfunction of intercellular contacts causes severe imbalance of ionic and metabolic homeostasis in the neuroglial network, significantly exacerbating secondary brain injury and contributing to the progression of pathological processes after TBI [57,58].

In the present study, the collagen matrix with Na_2_S_2_O_3_ most effectively preserved Cx43 levels in the acute phase and limited its hyperexpression and aggregation at later stages, correlating with reduced disorganization of the astrocytic cytoskeleton and decreased diffuse extracellular GFAP signal. In contrast, AOAA exacerbated all reactive changes.

Our molecular dynamics results also demonstrate that ischemic conditions, including decreased pH and increased Ca^2+^ concentration, lead to significant changes in the conformational dynamics of Cx43, particularly within the cytosolic domain, which plays a key role in regulating hemichannel gating [59]. The increased sensitivity of Cx43 to intracellular pH changes is consistent with experimental data. For example, it has been shown that pH alterations affect the conformation of the C-terminal region of Cx43, thereby modulating its channel-opening capacity. Studies of interactions between the C-terminal domain and cytoplasmic regions of the protein confirm that acidification can induce structural changes in Cx43, leading to impaired intercellular communication [60].

Moreover, previous studies have demonstrated that truncation of the C-terminal domain of Cx43 significantly reduces channel sensitivity to pH changes, highlighting the importance of this domain for channel function under acidic conditions [61]. It has also been shown that peptides encompassing regions 261–300 and 374–382 play a key role in pH-dependent regulation of channel opening, and mutations in these regions can impair the ability of Cx43 to mediate pH-dependent cell uncoupling, which may be associated with various pathological conditions [62]. These findings confirm that conformational changes in Cx43, particularly in response to pH variations, represent an important mechanism in the regulation of intercellular communication under both physiological and pathological conditions.

In the present study, it was shown that a decrease in pH leads to reduced mobility of the N-terminal domain of Cx43 (amino acid residues 1–18), which is involved in the formation of the hemichannel gating mechanism, and also affects the conformational dynamics of the C-terminal cytosolic domain (region 331–344). It is known that intracellular domains of Cx43, including the N-terminus, cytoplasmic loop, and C-terminus, form a coordinated regulatory system controlling the opening and closing of gap junction channels in response to intracellular environmental changes. In particular, the N-terminus is involved in the formation of the channel gating structure and plays a key role in transitions between open and closed states [62].

Such conformational changes have previously been associated with pH-dependent closure of Cx43 channels and reduced intercellular transport of ions and small metabolites, as acidosis induces interaction between the C-terminal domain and the cytoplasmic loop of the protein, implementing a “ball-and-chain” regulatory mechanism [60,62]. It is also well established that both intracellular pH and Ca^2+^ concentration are key chemical factors regulating gap junction permeability and their ability to mediate intercellular signaling [63].

The partial stabilization of Cx43 structure observed in our simulations in the presence of Ca^2+^ is consistent with known Ca^2+^-dependent regulation of Cx43, as increased intracellular Ca^2+^ concentration is one of the key factors leading to reduced gap junction conductance [64,65,66]. For example, in HeLa cells, elevation of intracellular Ca^2+^ levels induced by the Ca^2+^ ionophore ionomycin inhibits intercellular transfer of fluorescent dyes, indicating Ca^2+^-mediated reduction in Cx43 conductance [64]. A number of studies suggest that this effect may be mediated through Ca^2+^-dependent activation of calmodulin and subsequent regulation of the conformational state of connexin cytosolic domains, leading to channel closure and functional uncoupling of cells [67,68]. Furthermore, it has been shown that decreased pH can enhance Ca^2+^-mediated inhibition of intercellular communication by increasing intracellular Ca^2+^ levels [67].

It should be noted that in the present study, the effect of Ca^2+^ ions was modeled in the absence of calmodulin, allowing consideration of the possibility of direct Ca^2+^ action on connexin structural organization. Molecular dynamics simulations and mutational analyses have demonstrated that Ca^2+^ can interact with charged amino acid residues near the entrance of the hemichannel pore, disrupting salt bridge networks and stabilizing the closed state of the channel [69]. Such electrostatic interactions are thought to alter the mobility of functionally important domains of Cx43 and influence the probability of channel opening. Thus, the stabilization of specific regions of the cytosolic domain of Cx43 observed under simulated ischemic conditions in this study may reflect the combined effects of Ca^2+^ and pH on the conformational dynamics of a protein critically involved in the regulation of intercellular communication following neural tissue injury.

Analysis of the interactions between sulfur-containing ligands and Cx43 showed no formation of stable noncovalent complexes for HS^−^ and H_2_S within the simulation time frame. This is likely due both to the physicochemical properties of H_2_S as a small neutral molecule with predominantly weak and transient noncovalent interactions, and to the structural features of Cx43. The cytosolic domains of Cx43 are characterized by high conformational flexibility and the absence of well-defined hydrophobic pockets that facilitate stable ligand binding, which may limit the ability of small molecules to remain associated with potential interaction sites. In addition, many functionally significant cysteine residues of Cx43 are located in regions whose accessibility may strongly depend on connexin oligomerization, membrane interactions, and formation of the full hexameric hemichannel.

However, this result does not exclude a regulatory role of H_2_S since its biological effects are often mediated not through classical ligand–receptor interactions but via covalent post-translational modifications of proteins, primarily S-sulfhydration of cysteine thiol groups [19]. This mechanism represents an important component of the complex network of H_2_S-dependent biological signaling, leading to changes in protein conformational mobility, interactions with targets, and functional activity [70].

The most pronounced interactions in the simulations were observed for the S_2_O_3_^2−^ anion, which demonstrated the possibility of electrostatic interactions with positively charged arginine residues in the cytosolic domain of Cx43. The ability of polyvalent anions to form stable complexes with proteins has been described in various systems and is considered a potential mechanism of allosteric regulation of protein function [71,72]. The weakening of S_2_O_3_^2−^ interaction with Cx43 in the presence of Ca^2+^ may be explained by competitive binding of cations to negatively charged regions of the protein.

Modeling of the conformational dynamics of p53 revealed increased mobility of its intrinsically disordered domains under conditions of acidosis and in the presence of Ca^2+^, which may reflect reduced stability of the active form of the protein. It is well known that the structural integrity of the DNA-binding domain of p53 critically depends on Zn^2+^ coordination, which stabilizes the spatial organization of the protein [73]. Loss of Zn^2+^ or disruption of the coordination center leads to decreased DNA-binding affinity and altered transcriptional activity of p53, potentially promoting a shift toward transcription-independent functions, including involvement in mitochondrial apoptotic pathways.

Our data demonstrate that under conditions of acidosis and elevated Ca^2+^, retention of Zn^2+^ within the coordination center of p53 is weakened, which may contribute to destabilization of its active conformation. Importantly, in our simulations, Zn^2+^ was not retained in the coordination site of p53 under any conditions, which likely indicates more complex mechanisms of Zn^2+^ binding that may occur within higher-order p53–DNA complexes, similar to those observed in zinc finger proteins [74]. This is consistent with previous findings showing that, under physiological conditions, a significant fraction of p53 may exist in a Zn-free (apo-p53) state [75].

It is likely that Zn^2+^ serves as a key factor in conformational stabilization of the DNA-binding domain required for sequence-specific DNA interactions; in the absence of Zn^2+^, the protein becomes incapable of efficient DNA binding, while its structure becomes more flexible and prone to unfolding or aggregation. In the absence of Zn^2+^, p53 fragments exhibit increased flexibility and loss of the ability to stabilize critical loops within the DNA-binding domain, as supported by molecular dynamics and biophysical studies of Zn^2+^ in p53 structure [73]. Zn^2+^ delivery to intracellular proteins is likely mediated by specialized proteins capable of binding, transporting, and releasing transition metal ions depending on the cellular microenvironment. Such proteins include metallothioneins—low-molecular-weight cysteine-rich proteins that play a key role in maintaining Zn^2+^ homeostasis [76]. Regulation of intracellular Zn^2+^ levels by metallothioneins has been shown to act as an additional mechanism controlling apoptotic signaling and cellular stress responses [77]. The results obtained in this study expand current understanding of the complex, multilevel regulation of p53 activity, involving the ionic microenvironment, cellular redox state, and availability of cofactors.

We also demonstrated that sulfur-containing ligands are capable of interacting with p53 and altering the energetic characteristics of its conformational state. HS^−^ and H_2_S exhibit moderate Coulomb and van der Waals interactions with p53, indicating the possibility of transient binding to its surface. In contrast to Cx43, the protonated form of p53 shows a more pronounced tendency to interact with the S_2_O_3_^2−^ anion, as evidenced by significantly higher Coulomb energy values. This is likely due to the ability of divalent anions to form stable electrostatic interactions with positively charged amino acid residues of the protein [78]. The most stable interactions of S_2_O_3_^2−^ were observed in the p53 domain comprising residues 290, 320, and 357, suggesting possible effects on local conformational dynamics of p53. Considering the experimentally demonstrated ability of H_2_S to regulate p53 activity and reduce apoptosis under ischemic and TBI conditions, it can be assumed that S_2_O_3_^2−^ may influence the functional state of p53 through modulation of its structural organization.

The results of the in silico study complement the experimental findings and indicate the potential ability of sulfur-containing compounds to modulate the structural dynamics of Cx43 and p53 under ischemic conditions. The combined effects on intercellular communication via Cx43 and regulation of cell death via p53 may represent one of the mechanisms underlying the neuroprotective action of H_2_S donors in TBI.

In addition, it should be emphasized that the molecular dynamics simulations performed in the present study were intended primarily as an exploratory and hypothesis-generating approach for analyzing possible conformational changes and interactions of Cx43 and p53 under simulated ischemic conditions, which represent a characteristic process developing following TBI. The obtained in silico results were not directly validated by biochemical or biophysical experimental methods and therefore should not be interpreted as definitive evidence of the molecular mechanisms underlying the observed biological effects. Instead, the modeling data are intended to complement the morphological and immunohistochemical findings and to provide a theoretical basis for future studies involving direct experimental validation.

Thus, the present study demonstrates that a collagen matrix derived from *R. pulmo* and modified with Na_2_S_2_O_3_ represents a promising bioengineered platform that combines structural support for damaged tissue with potential modulation of key mechanisms of secondary brain injury. Both experimental observations and modeling data suggest that local administration of Na_2_S_2_O_3_ is associated with reduced p53 accumulation and apoptosis-related changes, partially preserves Cx43 expression, reduces neuroinflammation, and preserves the ultrastructure of nervous tissue, whereas inhibition of endogenous H_2_S synthesis leads to opposite effects. The modeled molecular interactions between sulfur-containing compounds and p53 and Cx43 expand current understanding of neuroprotective mechanisms and suggest their possible involvement in modulation of the cellular response to injury. Collectively, these findings support the potential application of modified marine-derived collagen matrices as next-generation biodegradable implants for the treatment of severe TBI, capable not only of replacing the defect but also of potentially influencing processes associated with secondary brain injury.

## 4. Materials and Methods

### 4.1. Ethical Approval

For the study of TBI, adult male CD-1 mice aged 14–15 weeks with a body weight of 20–25 g were used. The animals were housed in groups of 6–7 individuals in spacious cages, providing free access to food and water. The vivarium maintained optimal conditions: a temperature of 22–25 °C and effective ventilation with 18 air exchanges per hour, ensuring the comfort of the animals.

All experiments were conducted in strict accordance with international and national standards for the ethical treatment of laboratory animals. The studies complied with the provisions of Directive 2010/63/EU of the European Parliament and of the Council of 22 September 2010 on the protection of animals used for scientific purposes (available at: https://eur-lex.europa.eu/eli/dir/2010/63/oj (accessed on 22 May 2026)), as well as Russian regulations, including the “Rules of Laboratory Practice” (Ministry of Health of the Russian Federation Order No. 708n of 23 August 2010), and GOST 33215-2014 (Guidelines for Accommodation and Care of Laboratory Animals. Rules for Equipment of Premises and Organization of Procedures; available at: https://docs.cntd.ru/document/1200127789 (accessed on 22 May 2026)), which regulates conditions for the housing and procedures involving animals. The research protocol No. 2 was approved by the Bioethics Committee of Don State Technical University on 17 February 2020, confirming its adherence to high ethical and scientific standards.

Collection of *R. pulmo* jellyfish and procedures related to collagen isolation and processing were additionally approved by the Local Ethics Committee of Don State Technical University (Protocol of the Local Ethics Committee of DSTU No. 1 dated 3 July 2024).

### 4.2. Subjects and Procedure

For the modeling of severe TBI, adult male CD-1 mice aged 14–15 weeks and weighing 20–25 g were used. The modeling was performed according to the standard protocol for the free-falling weight procedure on the mouse brain cortex. General anesthesia was induced by intramuscular injection of a combined mixture of Tiletamine hydrochloride and Zolazepam hydrochloride (Zoetis Inc., Parsippany, NJ, USA) at a dose of 25 mg/kg and Xylazine (2% solution of Xylazine Hydrochloride; Interchemie Werken “de Adelaar” B.V., Waalre, Netherlands) at a dose of 5 mg/kg. The adequacy of anesthesia was assessed by the absence of response to pain stimuli and the suppression of the corneal reflex. Before surgical intervention and collagen matrix implantation, the fur was shaved from the head region, and the skin was treated with an aseptic solution. After fixation of the animal’s head, a trepanation hole of 3 mm in diameter was made using a dental drill. A controlled impact was applied to the cortex with a 150 g metal rod, 2 mm in diameter, dropped from a height of 3 cm. The trepanation area was irrigated with sterile saline, and a sterile collagen matrix of 3 mm diameter was implanted into the zone of impact using sterile surgical forceps. Depending on the experimental group, the matrix was used either in its native form or pre-impregnated with Na_2_S_2_O_3_ as an H_2_S donor or with AOAA as an inhibitor of endogenous H_2_S synthesis. The wound was sealed with bone wax.

Postoperative analgesia was performed using meloxicam (5 mg/kg, subcutaneously), administered immediately after surgery and subsequently once daily for the following two days [79,80]. During the postoperative period, the animals were monitored daily with assessment of general behavior, motor activity, and food and water intake, as well as signs of pain, distress, or neurological impairment. Humane endpoints included severe motor dysfunction preventing independent access to food and water, persistent seizure activity, body weight loss exceeding 20% of the initial weight, and signs of severe distress unresponsive to supportive care [81]. In such cases, the animals were immediately removed from the experiment and euthanized. All procedures related to anesthesia, analgesia, postoperative monitoring, and humane endpoints were approved by the Bioethics Committee of DSTU as part of the approved experimental protocol.

### 4.3. Collection of R. pulmo Jellyfish

Jellyfish *R. pulmo* were collected from the waters of the Azov Sea in August 2024 by hand. The selected biomaterial was transported in containers with chilled seawater (8 °C). For further research, sexually mature individuals with no signs of decay were selected, and their tentacles and intestinal cavities were removed beforehand. Before freezing, visible mechanical contaminants were removed by briefly rinsing the jellyfish with tap water, and the samples were then stored at −20 °C until collagen extraction.

### 4.4. Collagen Extraction from Jellyfish

Collagen extraction from *R. pulmo* jellyfish was performed by acid extraction using acetic acid. Before extraction, the jellyfish tissues were thoroughly rinsed with tap and distilled water. The tissues were then frozen and thawed three times over a period of 3 h, after being chopped into 2 × 2 cm pieces. The thawing process was done in a 0.1 M NaOH solution (BioLoT, Saint Petersburg, Russia), which helped to remove moisture, pigments, fats, and non-collagenous proteins. After the final thawing cycle, the jellyfish fragments were rinsed with distilled water and neutralized with 1% HCl. For collagen extraction, the tissues were ground with 0.5 M acetic acid in a 1:1 mass ratio, then diluted with 0.5 M acetic acid at a 1:3 volume ratio and kept at 4 °C for two days with continuous mixing. The mixture was filtered to separate the insoluble tissue fragments, which were then treated again with 0.5 M acetic acid, stirred at 4 °C for one hour, and filtered again.

The extract was centrifuged at 4 °C for 1 h to remove any remaining fine particles. The resulting supernatant was removed, and collagen was precipitated by gradually adding NaCl (Diaem, Moscow, Russia) to a final concentration of 0.9 M, with vigorous stirring. The collagen precipitate was collected by centrifugation at 10,000× *g* for 1 h at 4 °C.

Sterility was maintained by working in an aseptic environment, and solutions were prepared using deionized apyrogenic water. The collagen precipitate was dissolved in 0.5 M acetic acid and dialyzed to remove excess acid and low-molecular-weight substances. Dialysis was performed sequentially against 0.1 M, 0.05 M, and 0.025 M acetic acid for one day each, followed by dialysis against deionized apyrogenic water using a membrane with a molecular weight cutoff of 12 kDa. After dialysis, the collagen solutions were lyophilized in a Labconco FreeZone 2.5 L lyophilizer (Labconco Corporation, Kansas City, MO, USA) at −50 °C to produce porous sponges.

### 4.5. SDS-PAGE in Polyacrylamide Gel

The purity and type of collagen from *R. pulmo* jellyfish were evaluated by protein separation in polyacrylamide gel in the presence of sodium dodecyl sulfate (SDS) according to Laemmli’s method. For analysis, lyophilized collagen powder was dissolved at 4 °C for 12 h. Insoluble particles were removed by centrifugation at 10,000× *g* for 10 min, and the supernatant was collected for analysis. Protein content was measured using a SpectroSTAR Nano Plate Reader (BMG LABTECH GmbH, Ortenberg, Germany) and Bradford reagent (K002-500, Wuhan Fine Biotech Co., Ltd., Wuhan, China). Bovine serum albumin (BSA) was used as the standard. The obtained data were processed using MARS Data Analysis Software Ver. 3.33 (BMG Labtech GmbH, Ortenberg, Germany).

Buffer (62.5 mM Tris-HCl, pH 6.8, 10% glycerol, 2% SDS, 0.01% bromophenol blue, 5% β-mercaptoethanol) was mixed with prepared samples in a 1:1 ratio, and the sample was incubated at 70 °C for 10 min. Electrophoresis was conducted in a mini-PROTEAN Tetra system (Bio-Rad Laboratories, Hercules, CA, USA) in a 7% polyacrylamide gel with SDS. Protein markers were Affinity Prestained Protein Ladder (KF8009, Affinity Biosciences Ltd., Changzhou, China). Electrophoresis was conducted for two hours, with the first 20 min at a constant voltage of 120 V, and the following 1.5 h at 160 V. The gels were stained overnight at 24 °C in the dark with Coomassie Blue solution (0.25% Coomassie R250, 40% ethanol, 9% acetic acid). The stained gels were washed repeatedly with distilled water for two days, and the results were analyzed after imaging on a high-resolution gel documentation system SH-Advance523 (Shenhua Science Technology Co., Ltd. (SHST), Shijiazhuang, Hebei, China).

### 4.6. Matrix Preparation

For implantation into the TBI area, collagen matrices were formed into porous hemostatic sponges for filling the post-traumatic defect and creating a supporting scaffold for tissue regeneration. Sponges, approximately 3 mm in diameter and 2 mm in height, were formed from collagen hydrogel (15 mg/mL) obtained from *R. pulmo* jellyfish via acid extraction. The hydrogel was cooled to 4 °C and poured into 10 mL Petri dishes (60 mm in diameter), then frozen at −20 °C for 2 h followed by lyophilization. As a result of lyophilic treatment at low temperature and reduced pressure, three-dimensional matrices with a homogeneous structure and pronounced porosity were formed, ensuring the necessary porous architecture of the collagen scaffold. Sterilization of the obtained samples was performed by ultraviolet radiation in a laminar box for 1 h on both surfaces of the sponges. Circular blanks of the required diameter were cut from the sponge material immediately before implantation under aseptic conditions.

### 4.7. Staining of Matrices

For fluorescence assessment and visualization of the structure of collagen matrices, the sponges were stained with three fluorescent DNA dyes: Hoechst 33342, Propidium Iodide, and Sytox Green.

Hoechst 33342 dye is widely used for staining both live and fixed cells. When combined with other dyes, Hoechst allows the simultaneous visualization of DNA and other cellular structures or specific proteins. Its excitation and emission spectral characteristics do not overlap with the spectral ranges of most commonly used low-molecular-weight fluorophores and fluorescent proteins that fluoresce in the green–red region. Hoechst emits bright blue fluorescence with a peak emission at 461 nm when excited with ultraviolet light at approximately 350 nm. This makes it ideal for use in fluorescent microscopy [82].

In scientific research, the dye SYTOX Green is commonly used for staining cells with compromised plasma membrane integrity. It is unable to penetrate intact cells, and its detection via fluorescence microscopy indicates a late stage of necrosis or apoptosis. SYTOX Green emits intense green fluorescence with an emission peak at around 523 nm when excited with green light at 488 nm [83].

Propidium iodide, an intercalating DNA dye, is used in research to detect dead cells, as it cannot penetrate cells with an intact membrane. Its interaction with double-stranded DNA forms intercalated complexes that enhance fluorescence. Propidium iodide can be excited within a wavelength range of 400–600 nm and emits fluorescence between 600 and 700 nm [84].

For staining, 1 mL of phosphate-buffered saline (PBS) was added to three adjacent wells of a 24-well plate. In the first well, 5 µL of Hoechst 33342 working solution (SB-G1127-1ML, Servicebio Technology, Ltd., Wuhan, China) was added; in the second, 100 µL of propidium iodide (P3566, Thermo Fisher Scientific, Waltham, MA, USA) solution; and in the third, 20 µL of SYTOX Green (S7020, Thermo Fisher Scientific, Waltham, MA, USA) solution. The contents of each well were carefully mixed by gentle pipetting until a homogeneous solution was obtained. Collagen sponges were placed one by one into each of the three wells using sterile surgical forceps and incubated in the dark for 10 min at 25 °C. After incubation, the collagen matrices were transferred to glass slides and covered with cover slips before undergoing fluorescence microscopy.

### 4.8. Confocal and Fluorescent Microscopy

For the localization of proteins Cx43 and p53 in the brains of mice 24 h and 7 days after TBI, the following method was applied. Animals were anesthetized, and perfusion was carried out through the right ventricle of the heart using a 4% paraformaldehyde (PFA) solution. The animals were fixed head-down for 2 h to ensure even penetration of the PFA. The extracted brain was additionally incubated in 4% PFA for 12 h to complete the fixation. After fixation, coronal sections of 0.4 cm thickness, including the necrotic zone caused by TBI, were prepared. Thin sections, approximately 20 µm in thickness, were obtained on a Leica VT 1000 S vibratome (Leica Biosystems, Nussloch, Germany), followed by incubation in 15% and 30% sucrose solutions for 1 h each for cryoprotection, then freezing at −80 °C.

Sections were washed with phosphate-buffered saline (PBS) and treated with a blocking solution containing 5% bovine serum albumin (BSA, Sisco Research Laboratories Pvt. Ltd., Mumbai, India) and 0.3% Triton X-100 (Sisco Research Laboratories Pvt. Ltd., Mumbai, India) for 1 h at room temperature to minimize non-specific antibody binding. Sections were then incubated for 48 h at 4 °C with primary antibodies: rabbit anti-Cx43 (1:100; E-AB-70097; Elabscience Biotechnology Inc., Houston, TX, USA) and mouse anti-GFAP (1:1000, SAB4200571, Sigma-Aldrich, St. Louis, MO, USA), or rabbit anti-p53 (1:100, PAA928Mu01, Cloud-Clone Corp., Wuhan, China). After several washes in PBS, sections were treated with secondary antibodies: for confocal microscopy, anti-rabbit IgG (H + L) Abberior STAR 635P (1:500, Abberior GmbH, Göttingen, Germany) and anti-mouse IgG (H + L) Abberior STAR 580 (1:500, Abberior GmbH, Göttingen, Germany). For fluorescent microscopy, Alexa Fluor 555-conjugated goat anti-rabbit IgG secondary antibodies (1:500; ab150078, Abcam, Cambridge, UK) were applied.

Negative controls were performed without primary antibodies. Neuronal and glial cell nuclei were stained using Sytox Green Stain (ThermoFisher Scientific, Waltham, MA, USA) at a dilution of 1:1000 in PBS for confocal microscopy or Hoechst 33342 for fluorescent microscopy. Sections were incubated with the dye for 20–30 min at room temperature in the dark to ensure specific nuclear staining. After staining, sections were washed three times in PBS to remove excess dye and reduce background signal. Samples were then mounted in antifade mounting medium (Abberior GmbH, Göttingen, Germany) to protect fluorescent signals from degradation during prolonged illumination and covered with cover slips to prevent drying and optimize visualization.

The study was conducted on an inverted confocal laser scanning microscope Abberior Facility Line (Abberior Instruments GmbH, Göttingen, Germany), which provides high resolution for the analysis of cellular structures. For 3D modeling, Z-scanning was performed with a step of 200 nm and a pixel size of 40 nm. Three-dimensional image reconstruction was performed using ImageJ software (version 1.54j, National Institutes of Health, Bethesda, MD, USA). For fluorescent microscopy, an Olympus BX53 microscope (Olympus Corporation, Tokyo, Japan) equipped with a high-resolution digital camera (EXCCD01400KPA, Hangzhou ToupTek Photonics Co., Ltd., Hangzhou, China) was used.

For quantitative assessment of p53 expression levels in fluorescent microscopy images, ImageJ software (version 1.54j, National Institutes of Health, Bethesda, MD, USA) was used. A rectangular region was selected for the entire field of view on each image, and the mean fluorescence intensity was measured. Similarly, the background intensity in a region free from specific signal was measured. The background intensity value was subtracted from the mean signal intensity:

$$Relative\;Fluorescence\;Intensity\;(\%)={Mean}_{signal}-{Mean}_{background}$$

The obtained values were expressed as percentages relative to the background intensity and used for statistical analysis.

Colocalization of NeuN and Hoechst was also assessed using ImageJ version 1.51r (http://rsb.info.nih.gov/ij/, accessed 10 February 2017) with the JACoP plugin. The degree of signal overlap was quantified by calculating the M1 coefficient.

For quantitative assessment of Cx43 immunoreactivity, confocal images obtained under identical acquisition settings were analyzed using ImageJ software (version 1.54j, National Institutes of Health, Bethesda, MD, USA). For each animal, six independent images from standardized regions of the ipsilateral perifocal cortex and corresponding regions of the contralateral hemisphere were analyzed. The mean fluorescence intensity of Cx43 was measured within selected regions of interest, and background fluorescence was determined in areas free of specific signal. Normalized fluorescence intensity of Cx43 was calculated as the ratio between mean signal intensity and background fluorescence intensity. The final value for each animal was obtained by averaging measurements from all analyzed images. In addition, quantitative analysis of Cx43-positive aggregates was performed manually within standardized regions of interest. Large clustered Cx43-positive structures were counted and used for subsequent statistical analysis.

### 4.9. Transmission Electron Microscopy

To study the ultrastructure of brain tissues in mice after TBI, fragments of the parietal cortex, including the damaged area and similar regions from the undamaged hemisphere, were processed as follows:

The samples were fixed in a 2.5% glutaraldehyde solution (Aurion, Eugene, OR, USA) to preserve cellular architecture. Then, they were washed with PBS to remove any remaining fixative and post-fixed in a 1% osmium tetroxide (OsO_4_) solution in phosphate buffer for 1.5 h to stabilize lipid structures.

To prepare for embedding in epoxy resin, the samples were dehydrated by sequential incubation in increasing concentrations of ethanol (from 50% to absolute ethanol). Then, three stages of propylene oxide treatment were performed to facilitate infiltration, followed by embedding the tissues in epoxy resin based on Epon-812, creating a firm matrix for sectioning.

Semi-thin and ultrathin sections were made using an EM UC 7 ultramicrotome (Leica, Wetzlar, Hesse, Germany) with an Ultra 45° diamond knife (Diatome, Biel/Bienne, Canton of Bern, Switzerland). Ultrathin sections were contrasted with uranyl acetate and lead citrate solutions to enhance the visualization of intracellular organelles and membranes.

Ultrastructural analysis was conducted using a JEM-1011 transmission electron microscope (Jeol, Akishima, Tokyo, Japan) at an accelerating voltage of 80 kV, providing high-resolution images to study changes in neurons and glial cells after TBI.

### 4.10. Hematoxylin and Eosin Staining

For histological analysis of brain tissues after TBI, sections were stained with hematoxylin and eosin (H&E). Paraffin sections were obtained using a rotary microtome. Paraffin blocks with fixed brain tissue were pre-cooled on a refrigeration unit for 30 min to improve cutting properties. Sections, 3–4 µm thick, were obtained using disposable blades after trimming the block (20 µm) to remove excess paraffin and expose the tissue. The sections were carefully transferred to a water bath at 42–43 °C to flatten them, after which they were mounted on slides and dried at room temperature for 24 h.

After preparing the sections, routine H&E staining was performed to visualize morphological changes in the brain tissue. The sections were deparaffinized in three changes in xylene, followed by rehydration in decreasing concentrations of ethanol. Hematoxylin staining was performed for 25 min, after which the sections were differentiated in 1% hydrochloric acid and washed with running water to remove excess dye. The sections were then counterstained with eosin for 10–15 s, after which they were quickly washed with distilled water to prevent over-staining. Dehydration was performed in increasing concentrations of ethanol, followed by clearing with xylene in two changes. The sections were mounted using a mounting medium (Vitragel, BioVitrum, Saint Petersburg, Russia) and cover slips.

After drying, the preparations were analyzed under a light microscope to assess structural changes, including signs of necrosis, edema, inflammation, and neuronal damage in the injury zone.

H&E staining allowed clear visualization of cell nuclei and cytoplasm, providing a detailed assessment of tissue morphology and identification of pathological changes characteristic of TBI. Standardized staining protocols were used to increase the accuracy of the analysis, minimizing variability between samples.

Quantitative analysis of histological brain sections stained with H&E involved comparing normal and pathologically altered cells in the damage zone after TBI. Cell counting was performed at 400× magnification in ten randomly selected fields of view. The percentage of pathological cells was calculated using the following formula:

$$P_{path}=\frac{N_{path}}{N_{total}}\times 100$$

In this formula, N_path_ represents the number of pathologically altered cells, and N_total_ represents the total number of cells in the field of view. The calculation was performed at 400× magnification.

The infiltration area was measured at 100× magnification using the following formula:

$$Infiltration\;Area\;Percentage=\frac{Infiltrated\;Area}{Total\;area}\times 100$$

This formula calculates the percentage of the infiltration area relative to the total tissue area. “Infiltrated Area” refers to the measured area of infiltration, and “Total Area” refers to the total area of the examined section. The resulting value is expressed as a percentage and quantitatively evaluates the extent of the inflammatory process.

### 4.11. Molecular Dynamics Simulation

Molecular dynamics simulations (MDSs) were performed using the GROMACS 2026.0 program [85]. The studied models were the Cx43 molecules (Uniprot: P17302) in a bilipid layer (generated using the packmol package) and the p53 protein (Uniprot: P04637). Using the H++ resource [86], the protonated forms of these proteins were obtained at pH 6.5. Therefore, three computational systems were constructed for each model: the physiological (natural state), acidic (protonated protein form at pH 6.5), and ischemic states (protonated protein form at pH 6.5 with an additional 10 Ca^2+^ ions). To identify binding sites for ligands (HS^−^, H_2_S, and S_2_O_3_^2−^), five molecules of each ligand were added to each system. The system was neutralized with Na^+^ and Cl^−^ ions. Preparation of each system for simulation involved energy minimization (Fmax < 100 kJ/mol), followed by several iterations of system equilibration at a pressure of 1 atm and temperature of 310 K. The final MDS was carried out for 200 ns in the AMBER force fields (FF19SB for proteins, OPC for water and ions, and GAFF2 for ligands).

### 4.12. Statistical Analysis

Statistical analysis was performed using one-way analysis of variance (ANOVA) followed by the Newman–Keuls post hoc test. Normality of distribution was assessed with the Shapiro–Wilk test, and homogeneity of variances was evaluated using the Brown–Forsythe test. When the assumptions of normality or homogeneity of variances were violated, the non-parametric Kruskal–Wallis test was applied instead. Survival analysis was performed using Kaplan–Meier curves, and differences between groups were assessed using the log-rank test. Categorical data were analyzed using the χ^2^ (chi-square) test. All data were analyzed in a blinded manner. Differences were considered statistically significant at *p* < 0.05. Results are presented as mean ± standard error of the mean (M ± SEM). Data processing was carried out using SigmaPlot 12.5 (Systat Software Inc., San Jose, CA, USA).

In addition, animals were randomly assigned to experimental groups using a simple randomization procedure performed prior to surgical interventions. Investigators performing histological, immunofluorescence, and ultrastructural analyses were blinded to group allocation during data acquisition and analysis. To ensure blinding, each tissue sample and microscopy image was assigned anonymized identifiers that did not contain information regarding experimental groups or treatment conditions. It should be noted that, due to the specific nature of the invasive procedures, the investigator performing the surgical manipulations, including implantation of the collagen matrices, was aware of the matrix composition during surgery. However, all subsequent stages of image acquisition, quantitative analysis, and statistical processing were conducted in a blinded manner.

The present study was designed primarily as an exploratory experimental investigation. Sample sizes for survival analysis (*n* = 8 per group) and histological/immunofluorescence analyses (*n* = 6 per group) were selected based on previously published studies employing comparable experimental TBI models, as well as ethical considerations aimed at reducing the number of laboratory animals used in accordance with the 3R principles [87]. A priori statistical power analysis was not performed prior to the initiation of the study. At the same time, the relatively limited sample size may have reduced the statistical power for detection of moderate biological effects, particularly in the survival analysis.

## 5. Limitations of the Study

Despite the promising results obtained, the present study has several limitations that should be considered when interpreting the data. First, the study was exploratory and pilot in nature, and the sample sizes used were relatively limited, which may have reduced the statistical power of the analysis, particularly for survival assessment and detection of moderate intergroup differences. Second, the experimental design did not include a sham-operated group involving craniotomy without cortical injury. Although all experimental groups underwent identical surgical procedures, including anesthesia, trepanation, and tissue manipulation, the absence of a sham control limits the ability to fully distinguish the contribution of surgical trauma, anesthesia, and post-traumatic changes to the observed neuroinflammatory and glial responses. In addition, the TEM analysis was primarily focused on groups with pharmacological modulation of H_2_S signaling. It should also be taken into account that the observed effects of Na_2_S_2_O_3_ may be related not only to its role as an H_2_S donor but also to the intrinsic biological and antioxidant properties of thiosulfate itself, which may independently influence the severity of post-traumatic alterations in nervous tissue. Therefore, the specific contribution of H_2_S-dependent mechanisms requires further clarification in additional experimental studies. It should also be considered that, due to the specific nature of the invasive surgical procedures, the investigator performing TBI modeling and matrix implantation was aware of the composition of the implanted matrices during surgery. However, all subsequent stages of morphological, immunohistochemical, confocal, and statistical analyses were performed using coded samples without information regarding experimental group allocation. Furthermore, although quantitative immunofluorescence analysis of Cx43 and p53 was performed, the present study did not include direct quantitative assessment of protein and gene expression using approaches such as Western blotting or quantitative PCR. In addition, no direct biochemical validation of the proposed molecular signaling pathways was performed. Therefore, the obtained data should be interpreted primarily as morphological, immunohistochemical, and exploratory observations requiring further molecular validation. Finally, despite the quantitative analysis of Cx43, the present study was primarily focused on morphological and immunohistochemical aspects of nervous tissue injury and did not include functional assessments such as electrophysiological parameters or long-term behavioral outcomes following TBI. Therefore, further studies are required to more comprehensively investigate the molecular mechanisms underlying H_2_S-dependent neuroprotection and to evaluate the long-term efficacy of the developed collagen matrix in traumatic brain injury.

## Figures and Tables

**Table 1:** Statistical characteristics of interaction energies between connexin 43 and ligands HS^−^, H_2_S, and S_2_O_3_^2−^. Reference—physiological model; Acidic—protonated model at pH 6.5; Ischemic—protonated model at pH 6.5 in the presence of Ca^2+^.

Name	Energy Average ± Err. Est. (kJ/mol)	RMSD	ETot-Drift (kJ/mol)
Coulomb interactions of Connexin with HS^−^ in different conditions			
Reference	−4.171 ± 0.53	19.08	−0.3025
Acidic	−5.128 ± 2.3	24.72	−10.9069
Ischemic	−8.364 ± 2.8	30.29	3.2124
Van der Waals interactions of Connexin with HS^−^ in different conditions			
Reference	−0.1616 ± 0.034	1.654	−0.08421
Acidic	0.04672 ± 0.11	2.316	0.5429
Ischemic	−0.1124 ± 0.04	2.680	0.06951
Coulomb interaction of Connexin with H_2_S in different conditions			
Reference	−1.8128 ± 0.79	5.299	1.551
Acidic	−5.5677 ± 2.9	9.536	14.22
Ischemic	−0.7728 ± 0.25	3.594	0.2990
Van der Waals interactions of Connexin with H_2_S in different conditions			
Reference	−3.413 ± 1.6	5.945	3.563
Acidic	−8.278 ± 2.4	7.976	9.605
Ischemic	−1.350 ± 0.35	3.486	0.9790
Coulomb interactions of Connexin with S_2_O_3_^2−^ in different conditions			
Reference	−284.2 ± 76	195	−441.0
Acidic	−377.8 ± 62	180.7	−315.4
Ischemic	−43.09± 15	69.70	−29.07
Van der Waals interactions of Connexin with S_2_O_3_^2−^ in different conditions			
Reference	10.13 ± 3	13.66	18.64
Acidic	17.43 ± 4.9	17.98	26.99
Ischemic	0.5473 ± 0.68	5.604	1.111

**Table 2:** Statistical characteristics of interaction energy changes between p53 and HS^−^, H_2_S, and S_2_O_3_^2−^. Reference—physiological model; Acidic—protonated model at pH 6.5; Ischemic—protonated model at pH 6.5 in the presence of Ca^2+^ ions.

Name	Energy Average ± Err. Est. (kJ/mol)	RMSD	ETot-Drift (kJ/mol)
Coulomb interactions of p53 with HS^−^ in different conditions			
Reference	−7.4516 ± 1.6	25.74	2.040
Acidic	−7.353 ± 2.1	27.55	8.682
Ischemic	−4.974 ± 1.3	21.63	−1.222
Van der Waals interactions of p53 with HS^−^ in different conditions			
Reference	−0.09209 ± 0.072	2.456	0.1380
Acidic	−0.02834 ± 0.12	2.530	−0.2424
Ischemic	−0.2008 ± 0.05	2.056	0.02648
Coulomb interactions of p53 with H_2_S in different conditions			
Reference	−2.106 ± 0.44	6.123	−1.999
Acidic	−1.148 ± 0.45	4.135	0.5842
Ischemic	−1.823 ± 0.19	5.698	0.5858
Van der Waals interactions of p53 with H_2_S in different conditions			
Reference	−3.306 ± 0.63	6.084	−1.568
Acidic	−2.385 ± 0.89	4.959	1.747
Ischemic	−3.116 ± 0.41	5.407	1.842
Coulomb interactions of p53 with S_2_O_3_^2−^ in different conditions			
Reference	−475.92 ± 110	294.7	626.5
Acidic	−932.95 ± 69	248.8	−322.9
Ischemic	−474.17 ± 47	180.0	−305.5
Van der Waals interactions of p53 with S_2_O_3_^2−^ in different conditions			
Reference	23.55 ± 2.7	20.48	−9.916
Acidic	55.51 ± 6.7	29.98	43.85
Ischemic	3.87 ± 1.1	16.92	1.571

## Data Availability

The original contributions presented in this study are included in the article/Supplementary Materials. Further inquiries can be directed to the corresponding author.

## Supplementary Materials

The following supporting information can be downloaded at: https://www.mdpi.com/article/10.3390/ijms27115134/s1.
