# Supplementary figures and images for: Development of an H2S-Associated Matrix Based on Rhizostoma pulmo Jellyfish Collagen: A Pilot Evaluation of Neuroprotective Effects and Cx43/p53 Regulation in Penetrating Traumatic Brain Injury
Source: Int J Mol Sci. 2026 Jun 5;27(11):5134. doi: 10.3390/ijms27115134 (PMC13257637; doi:10.3390/ijms27115134)

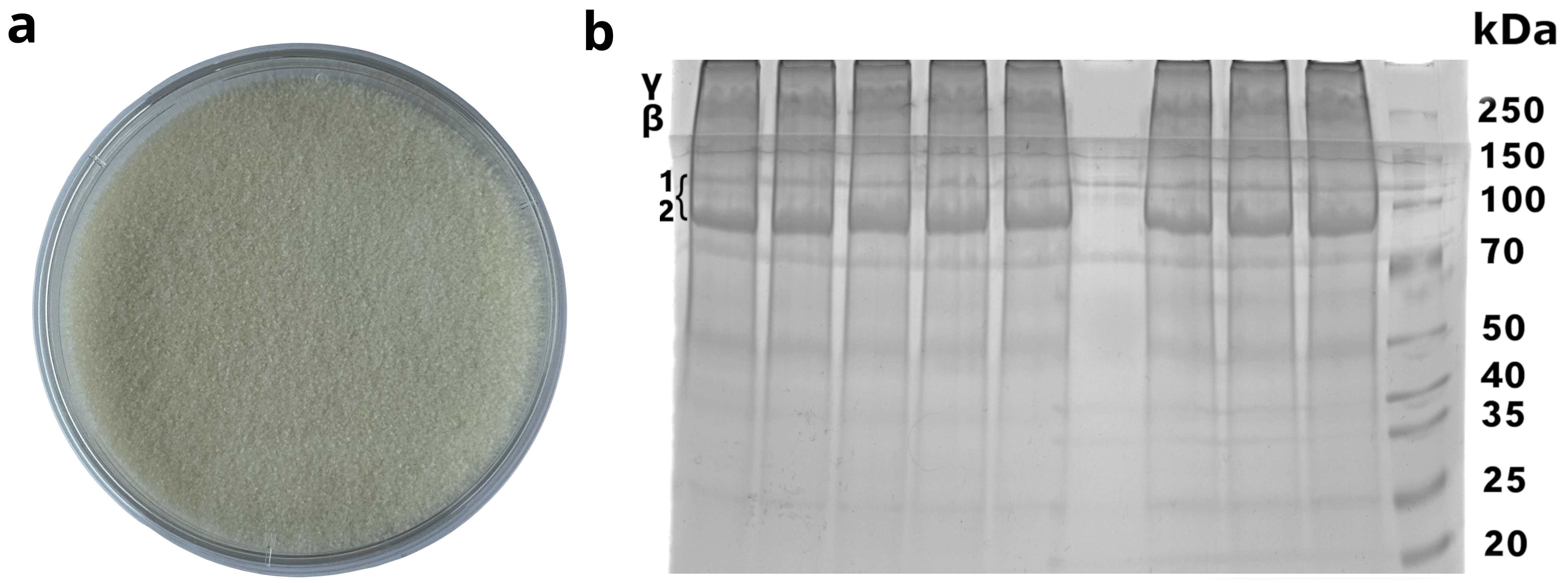

Supplement: Supplementary file 1 [file ijms-27-05134-s001.zip › Figure S1.tif]

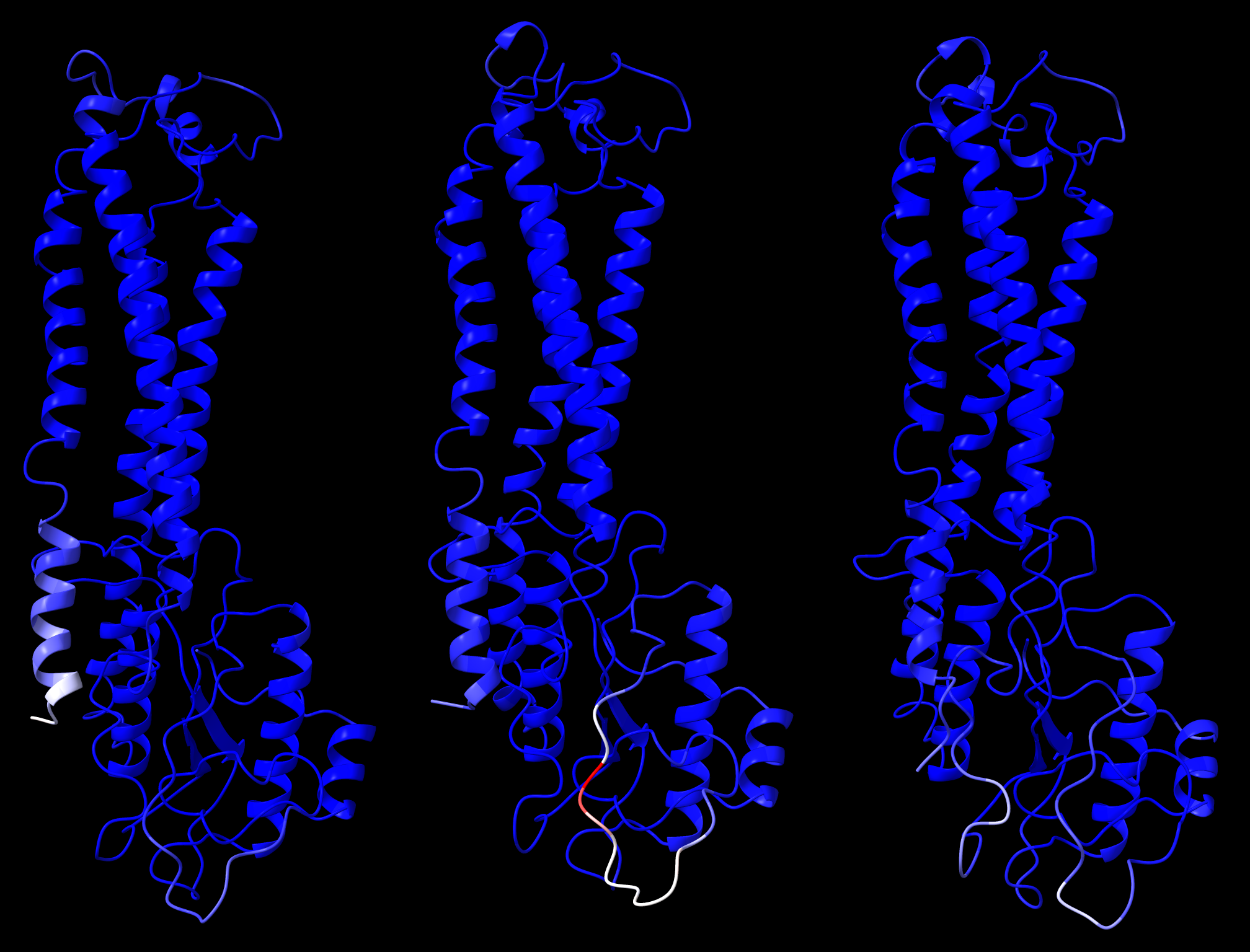

Supplement: Supplementary file 1 [file ijms-27-05134-s001.zip › Figure S10.png]

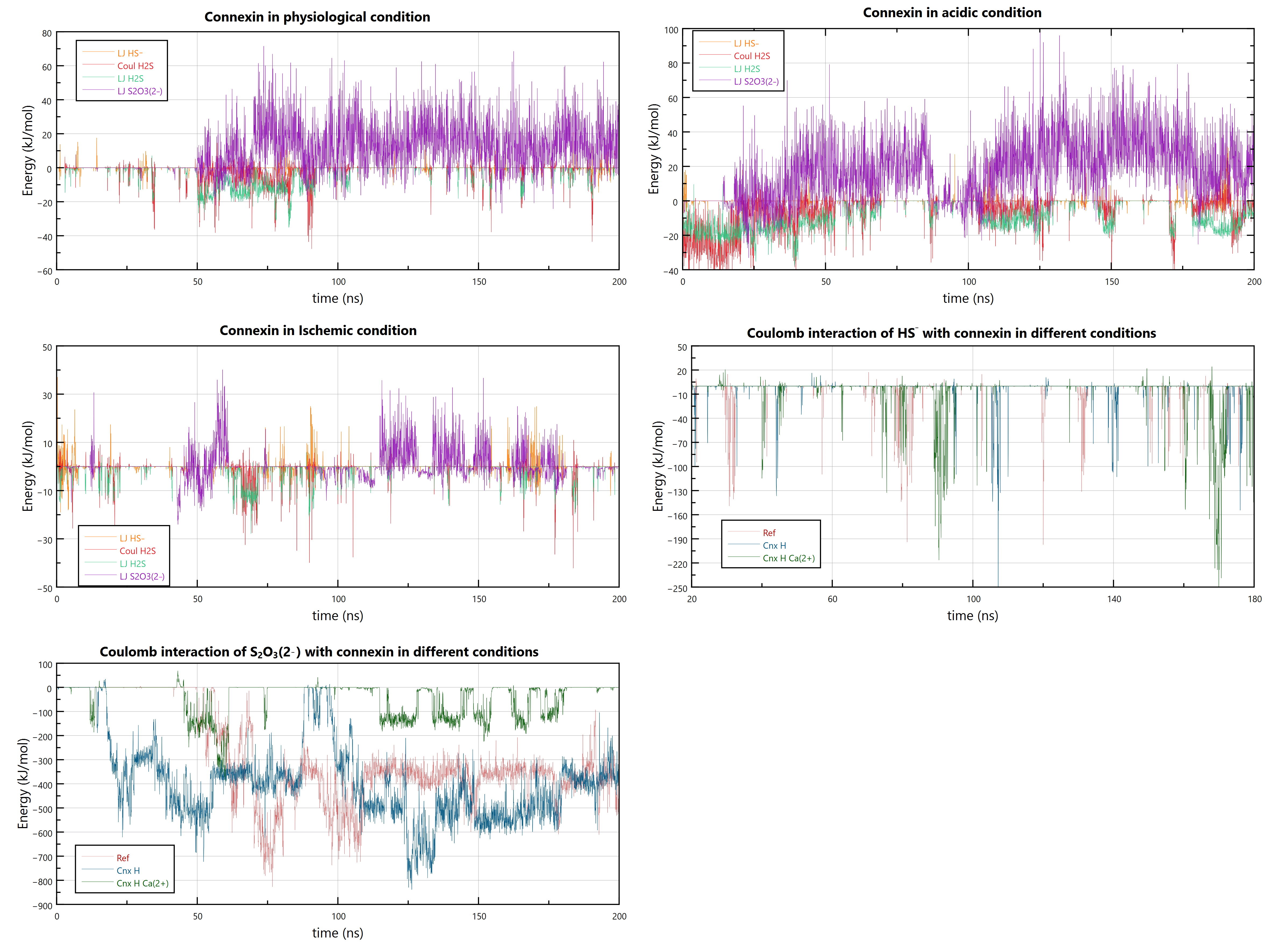

Supplement: Supplementary file 1 [file ijms-27-05134-s001.zip › Figure S11.tif]

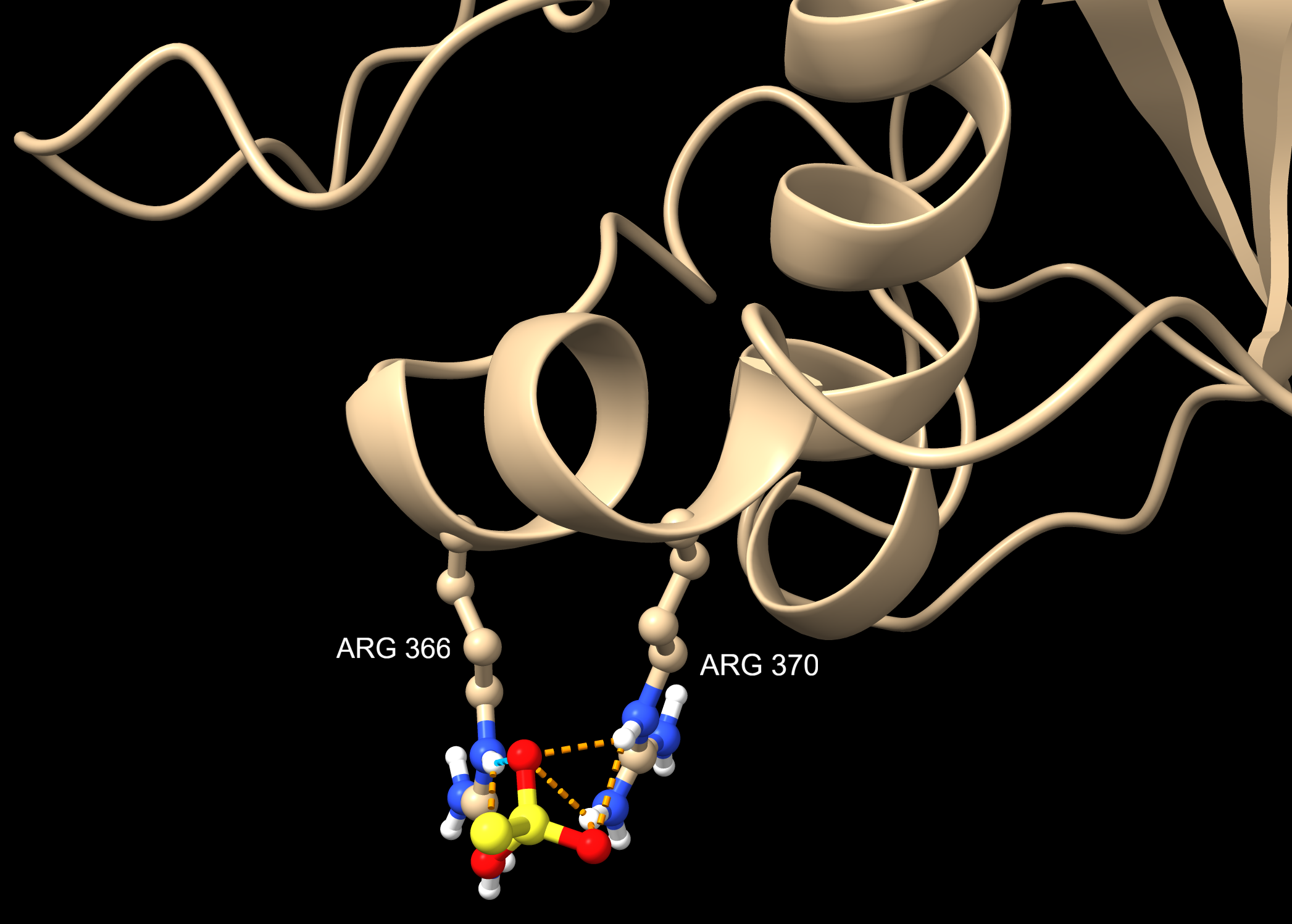

Supplement: Supplementary file 1 [file ijms-27-05134-s001.zip › Figure S12.png]

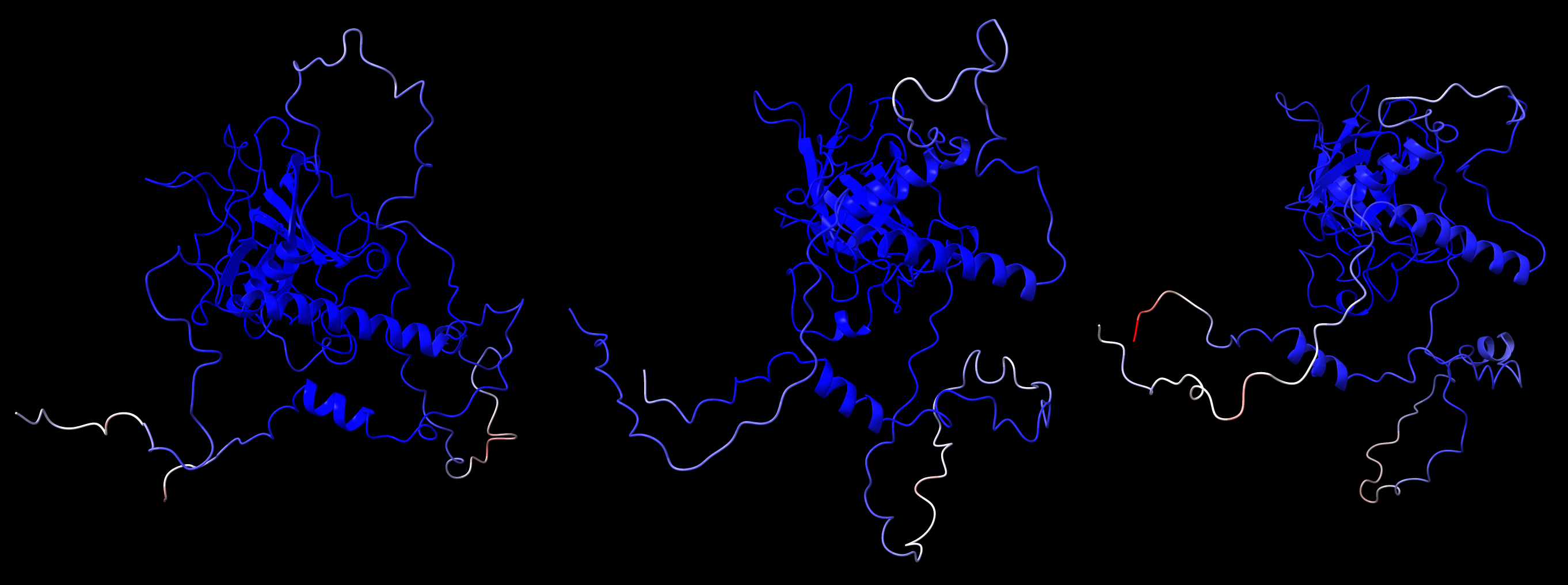

Supplement: Supplementary file 1 [file ijms-27-05134-s001.zip › Figure S13.png]

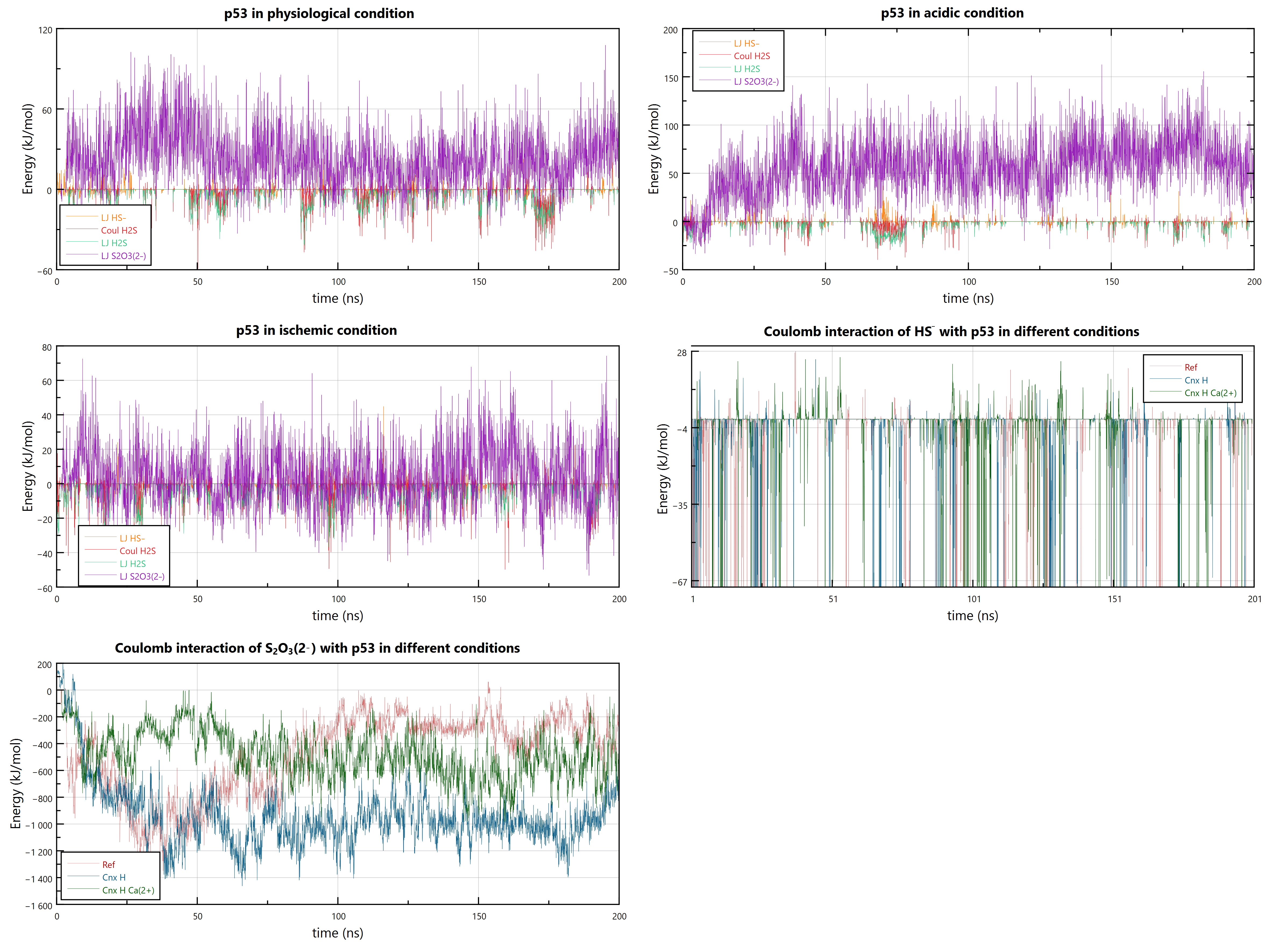

Supplement: Supplementary file 1 [file ijms-27-05134-s001.zip › Figure S14.tif]

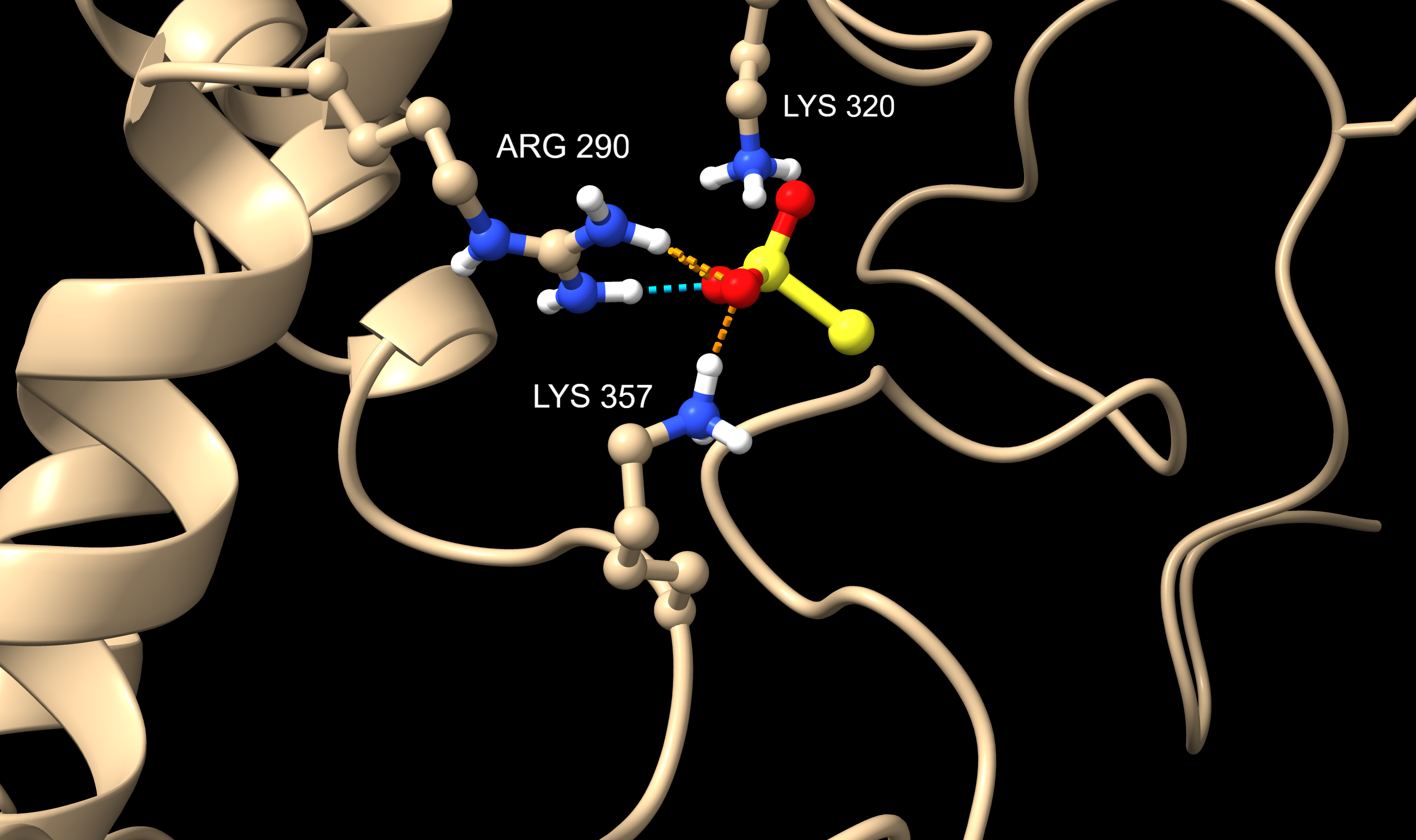

Supplement: Supplementary file 1 [file ijms-27-05134-s001.zip › Figure S15.png]

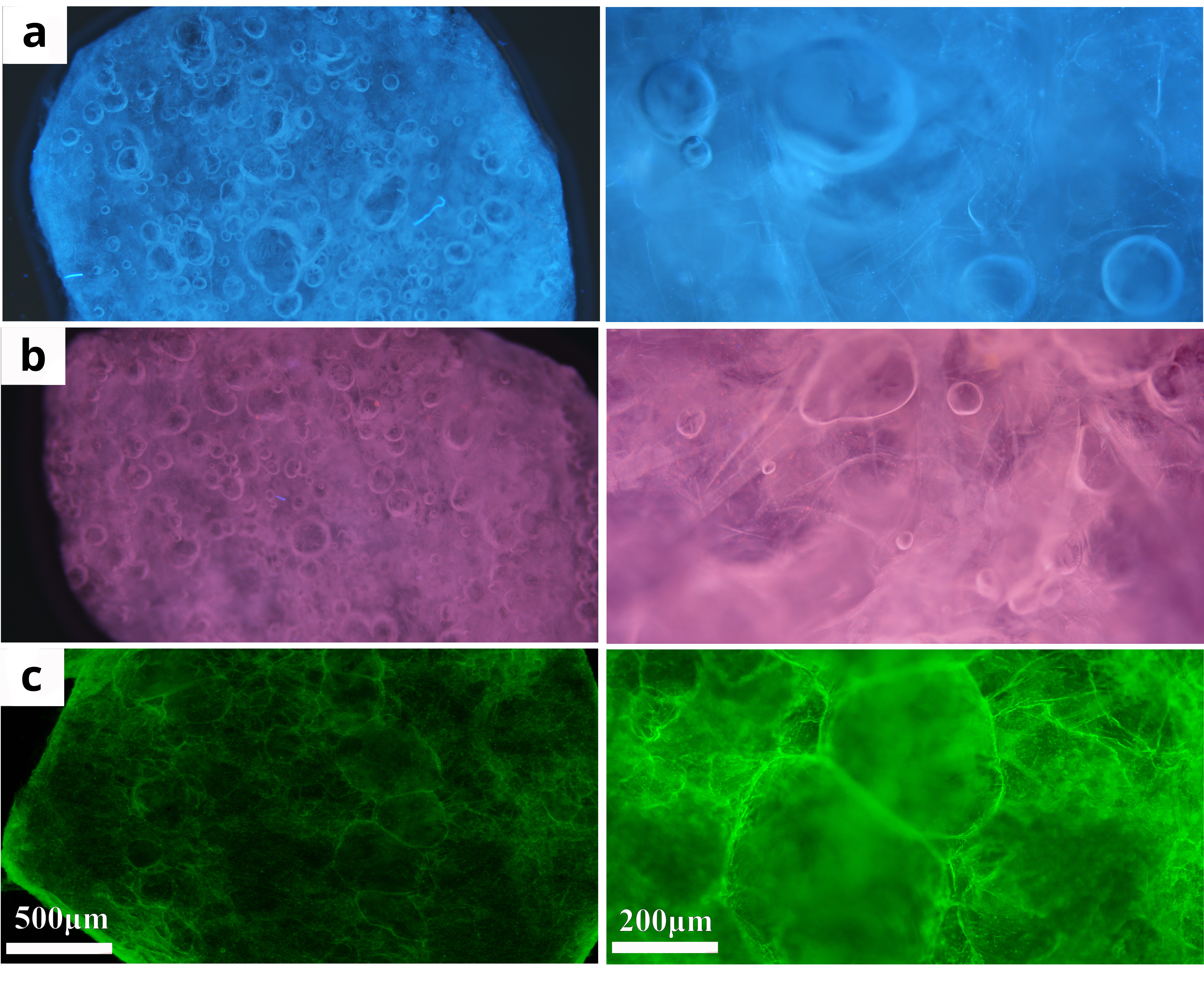

Supplement: Supplementary file 1 [file ijms-27-05134-s001.zip › Figure S2.tif]

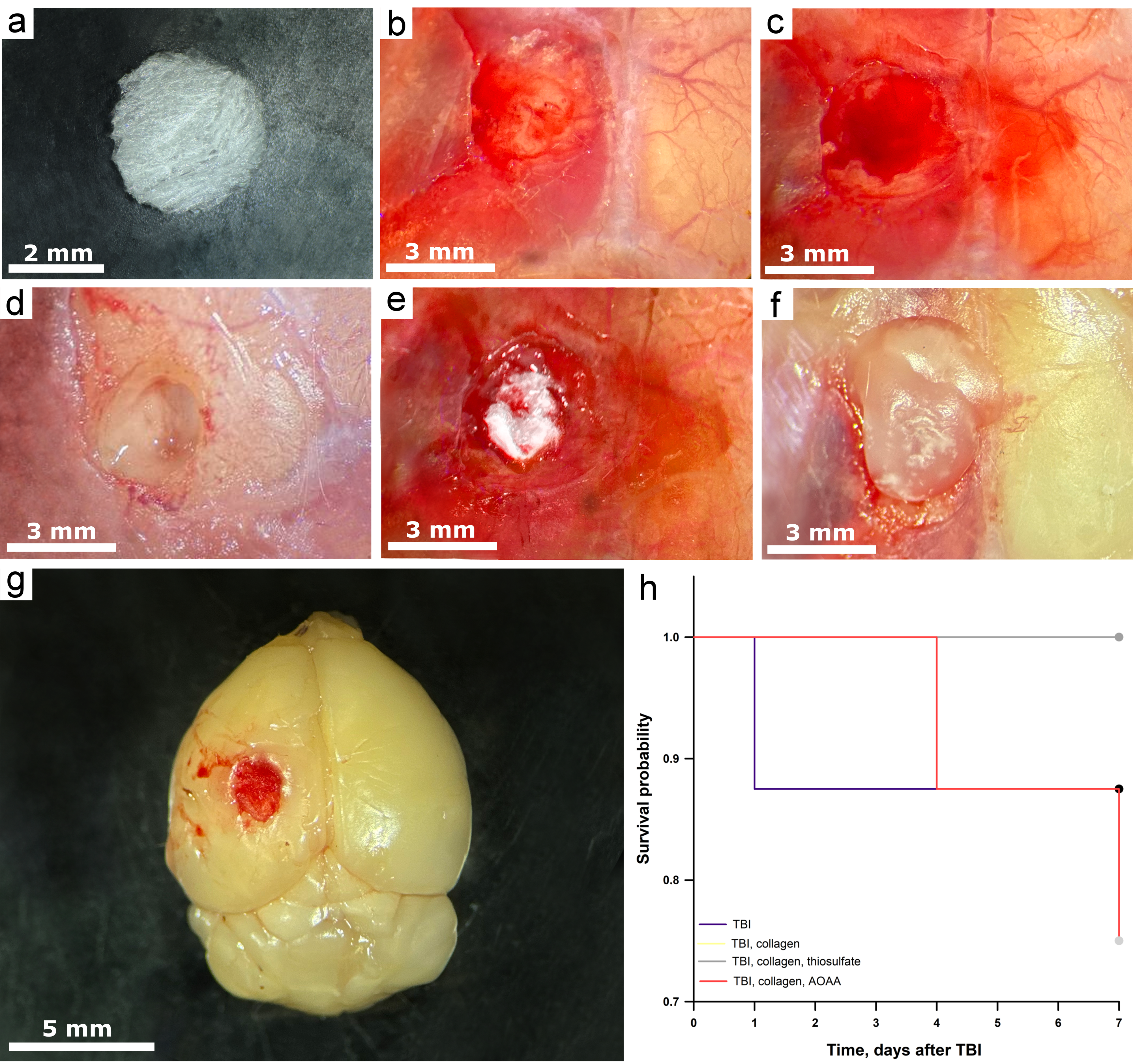

Supplement: Supplementary file 1 [file ijms-27-05134-s001.zip › Figure S3.tif]

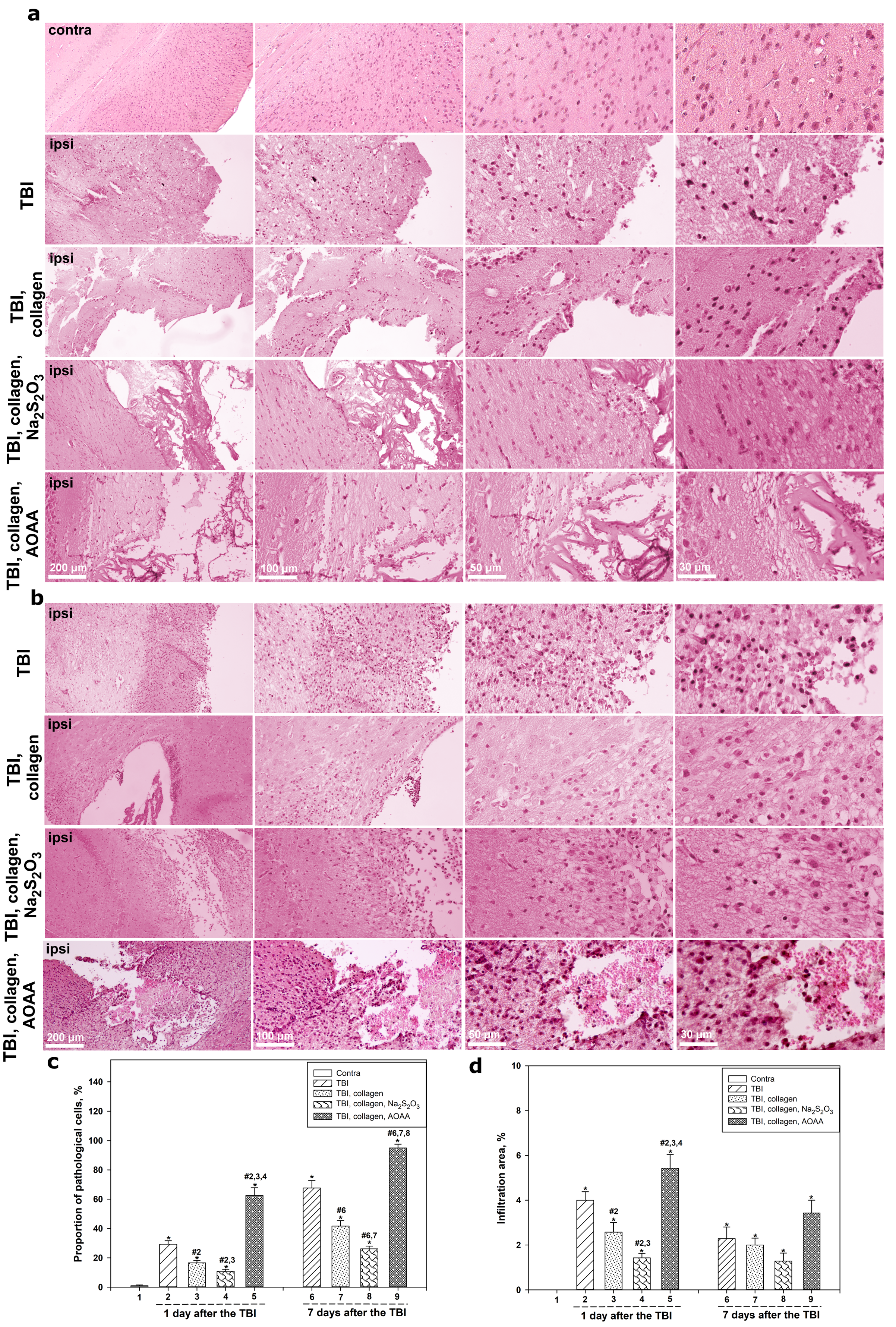

Supplement: Supplementary file 1 [file ijms-27-05134-s001.zip › Figure S4.tif]

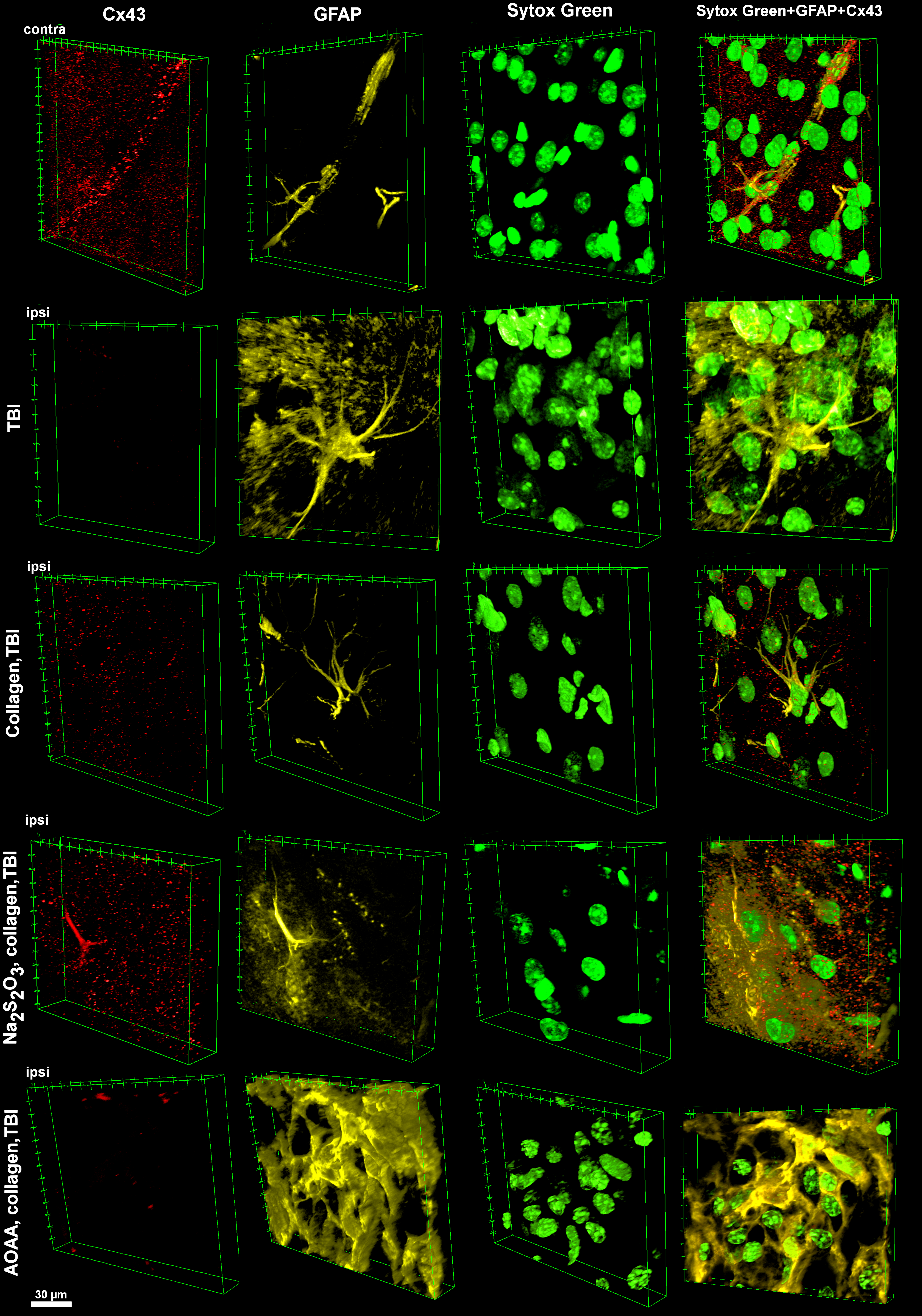

Supplement: Supplementary file 1 [file ijms-27-05134-s001.zip › Figure S5.tif]

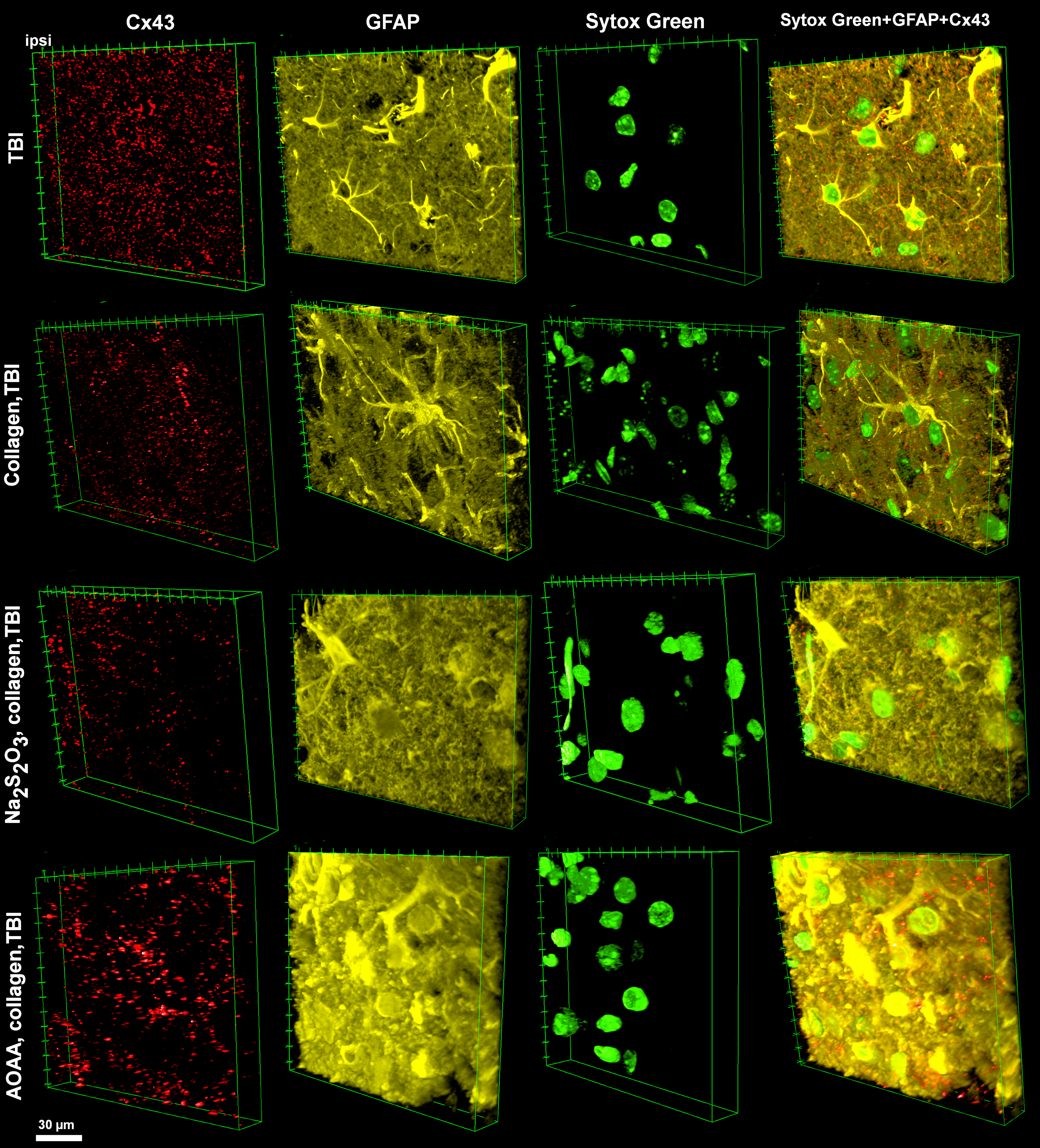

Supplement: Supplementary file 1 [file ijms-27-05134-s001.zip › Figure S6.tif]

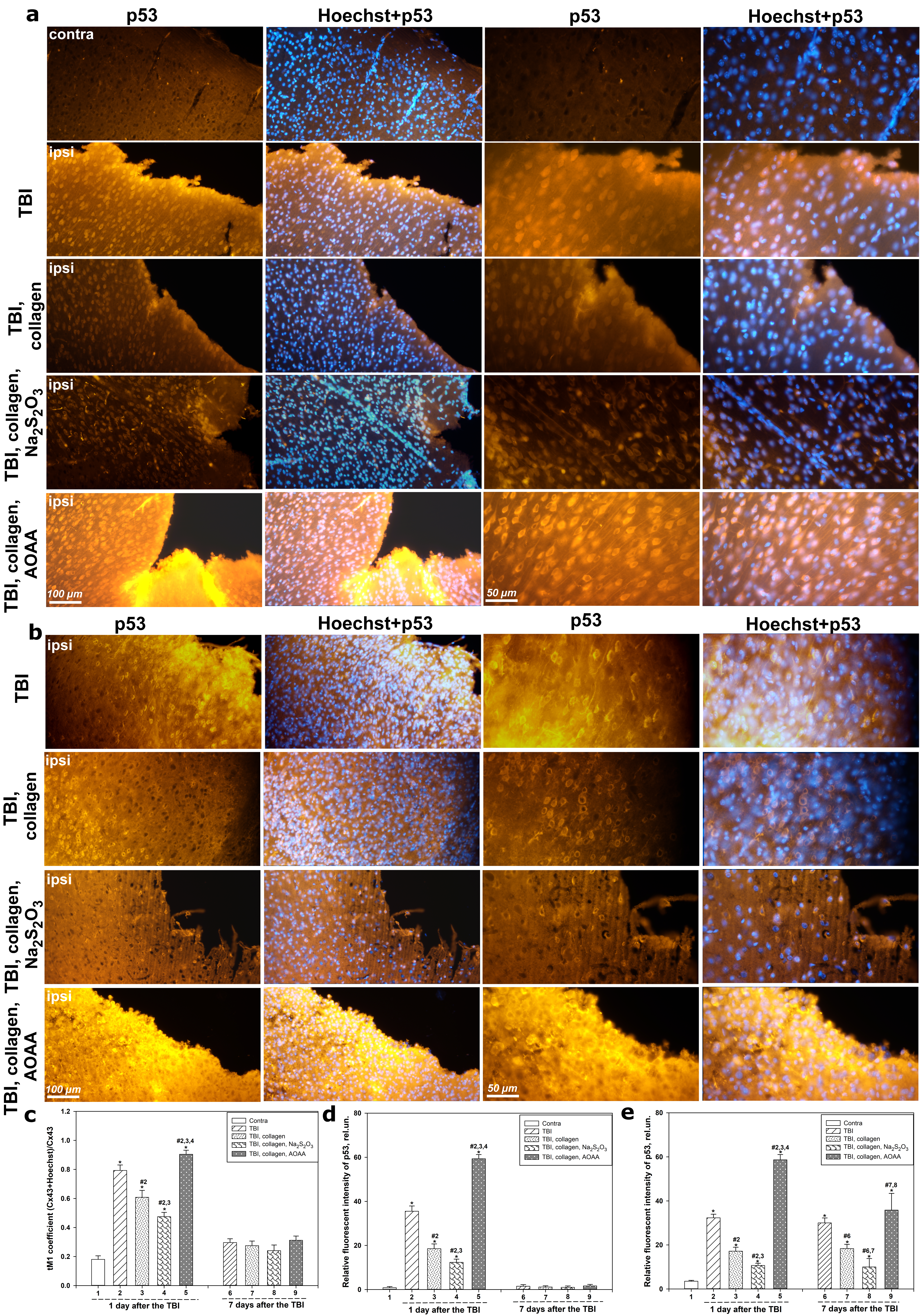

Supplement: Supplementary file 1 [file ijms-27-05134-s001.zip › Figure S7.tif]

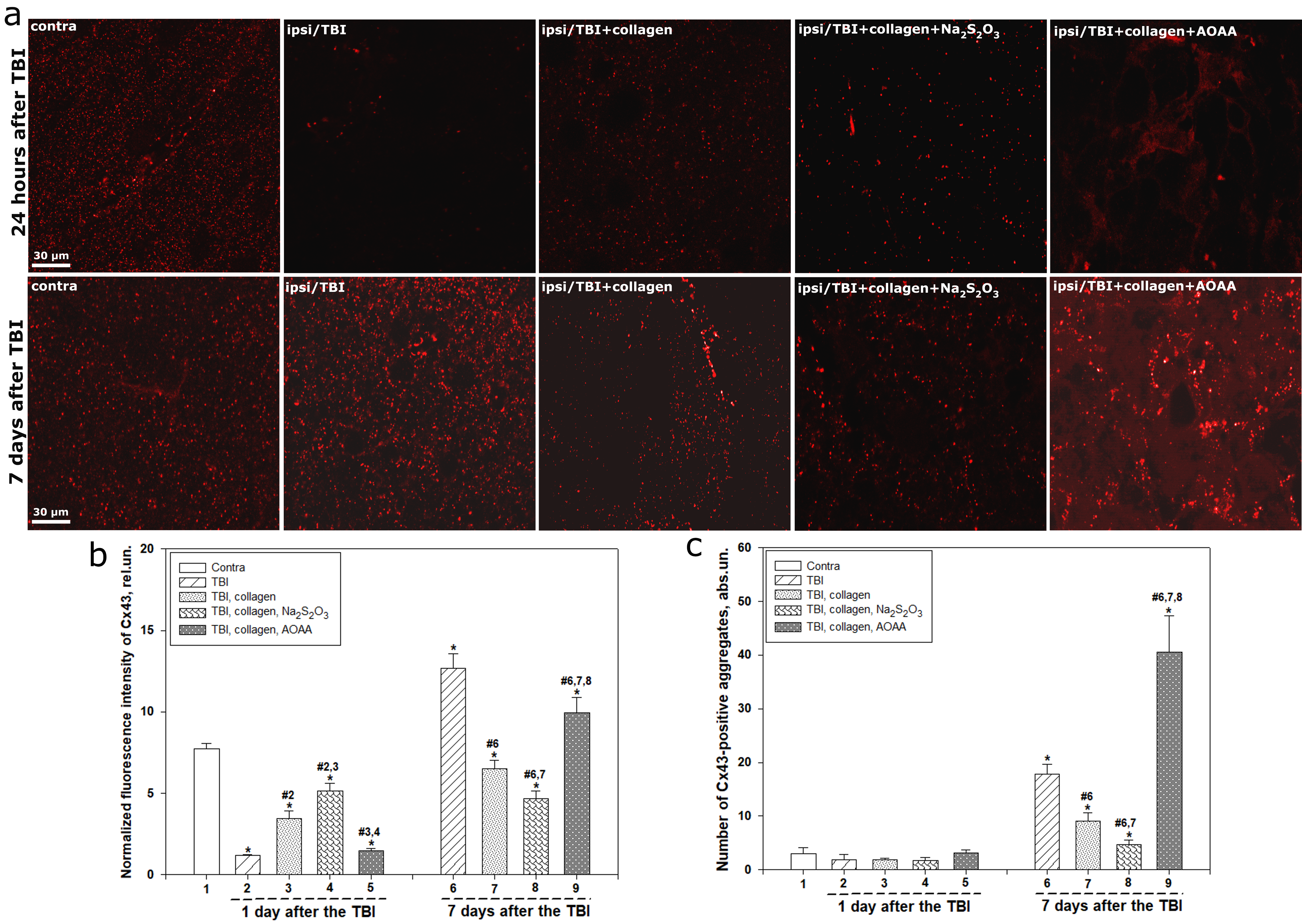

Supplement: Supplementary file 1 [file ijms-27-05134-s001.zip › Figure S8.tif]

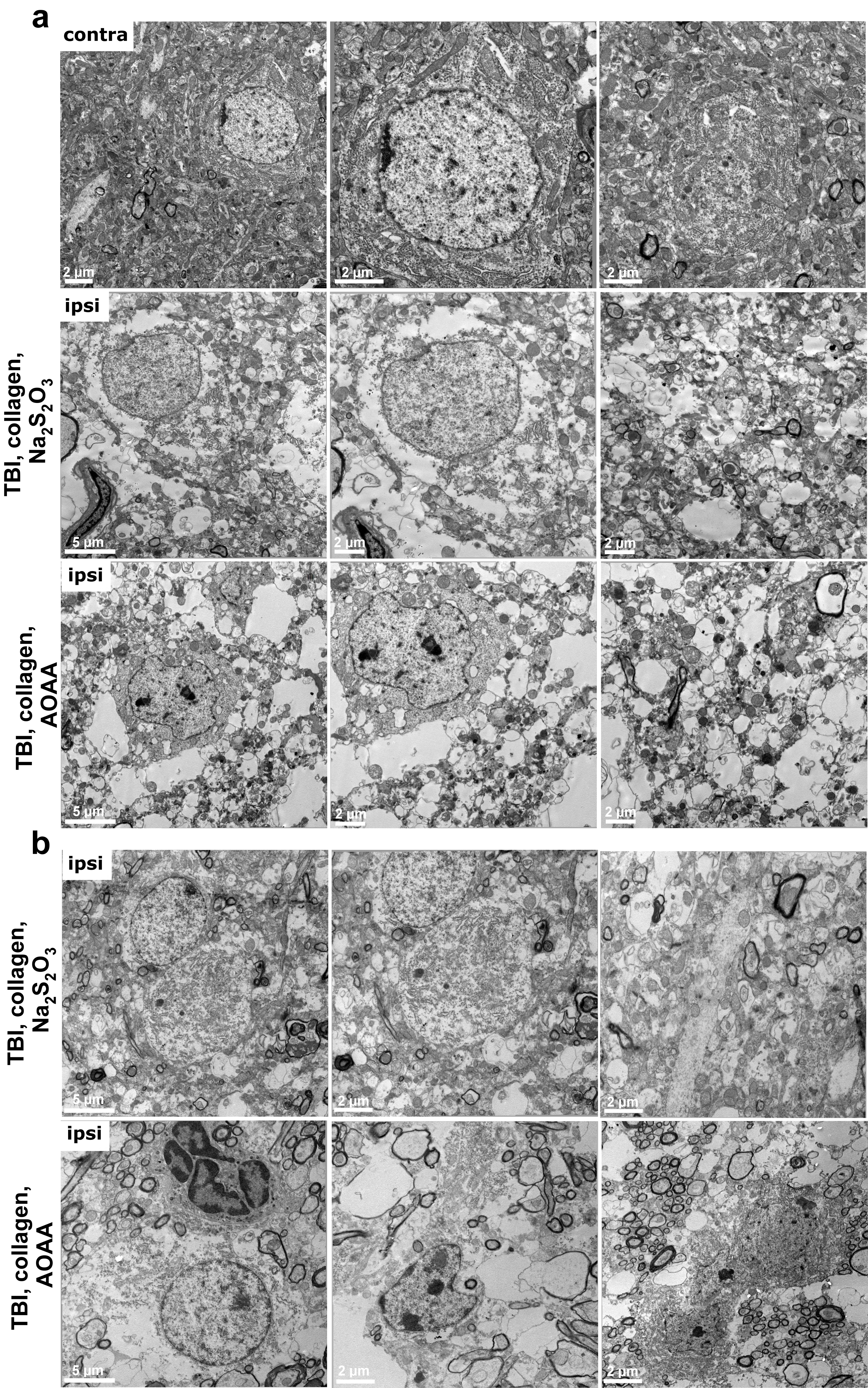

Supplement: Supplementary file 1 [file ijms-27-05134-s001.zip › Figure S9.tif]
